# MdBBX47, a B-box transcription factor directly and indirectly regulates ALA-induced anthocyanin accumulation in apple

**DOI:** 10.1186/s12870-026-08838-7

**Published:** 2026-04-29

**Authors:** Mohsin Iqbal, Liuzi Zhang, Yifan Yin, Zijing Yang, Jianting Zhang, Liangju Wang

**Affiliations:** College of Horticulture, Nanjing Agricultural University, Nanjing, 210095 China

**Keywords:** 5-Aminolevulinic acid (ALA), Anthocyanin accumulation, *Malus × domestica*, MdBBX47, *MdMYB110a*, Transcriptional regulation

## Abstract

The red pigmentation of apple (*Malus × domestica* Borkh.) fruit peel arises from the accumulation of anthocyanins. Anthocyanins, a pivotal determinant of fruit coloration, not only define commercial appeal but also confer nutritional and antioxidant benefits. 5-Aminolevulinic acid (ALA), a naturally occurring plant growth substance, has been shown to significantly intensify fruit redness by stimulating anthocyanin biosynthesis. Nevertheless, the underlying molecular mechanism remains largely unclear. In this investigation, we identified and functionally characterized MdBBX47, an ALA-responsive transcription factor whose transcriptional activation exhibits strong correlation with anthocyanin accumulation across apple fruits, leaves, and callus tissues, implicating it is a key regulatory node in the anthocyanin biosynthesis cascade. Subcellular localization assays confirmed that MdBBX47 is localized within the nucleus. Overexpression of *MdBBX47* substantially elevated the anthocyanin content, whereas RNA interference-mediated silencing markedly attenuated ALA-induced pigmentation, underscoring its positive regulatory function. Yeast one-hybrid (Y1H), luciferase (LUC), GUS, and electrophoretic mobility shift assays (EMSA) revealed that MdBBX47 directly binds to the promoters of *MdCHS* and *MdUFGT* to activate their expressions. Additionally, MdBBX47 can bind to the promoter of *MdMYB110a*, indirectly mediating anthocyanin accumulation. Collectively, these findings elucidate how MdBBX47 serves as a novel regulatory component involved in ALA-induced apple anthocyanin accumulation.

**Supplementary Information:**

The online version contains supplementary material available at 10.1186/s12870-026-08838-7.

## Core

ALA significantly promotes anthocyanin accumulation in apple, but the underlying mechanism remains unclear. Our results demonstrate that MdBBX47, as a key regulatory factor, plays a crucial role in mediating ALA-induced anthocyanin biosynthesis.

## Gene & accession numbers

The accession numbers are: *MdBBX47* (MD17G1119500), *MdCHS* (MD04G1003300), *MdF3H* (MD15G1246200), *MdDFR* (MD15G1024100), *MdANS* (MD06G1071600), *MdUFGT* (MD01G1234400), *MdGSTF12* (MD17G1272100), *MdMYB1* (MD09G1278600), *MdMYB9* (MD08G1070700), *MdMYB10* (MD09G1278400), *MdMYB110a* (MD17G1261000), *MdbHLH3* (MD11G1286900), *MdbHLH33* (MD07G1137500), *MdTTG1* (MD14G1031200), *MdMADS1* (MD17G1065400), *MdWRKY71* (MD10G1266400), *MdERF78* (MD15G1036500), *MdHsfB3a* (MD12G1079300), *MdHY5* (MD08G1147100) and *MdActin* (LOC103453508).

## Introduction

Apple (*Malus × domestica* Borkh.) represents one of the most extensively cultivated fruit crops globally, with red-skinned cultivars exhibiting significantly higher consumer preference compared to green or yellow variants. The distinctive red pigmentation of apple peel is predominantly attributed to the accumulation of anthocyanins, a class of hydrophilic flavonoid compounds that impart coloration including complex biosynthetic regulation. Beyond their critical role in determining fruit quality and market appeal, anthocyanins serve as bioactive phytochemicals with multifaceted physiological benefits, encompassing glycemic modulation, anti-senescence properties, and protective effects against diverse metabolic and degenerative disorders [1]. The anthocyanin biosynthetic pathway has been the focus of extensive research over recent decades [2]. Anthocyanins are synthesized through a branch of the phenylpropanoid pathways, which are highly conserved in plants. A sequential series of structural enzymes and their encoding genes are involved in, such as phenylalanine ammonia-lyase (*PAL*), chalcone synthase (*CHS*), chalcone isomerase (*CHI*), flavanone 3-hydroxylase (*F3H*), flavonoid 3’-hydroxylase (*F3’H*), flavonoid 3’,5’-hydroxylase (*F3’5’H*), dihydroflavonol 4-reductase (*DFR*), anthocyanidin synthase (*ANS*), and UDP-glucose: flavonoid 3-O-glucosyltransferase (*UFGT*) [3]. Furthermore, glutathione S-transferase (*GST*) and multidrug and toxin efflux (*MATE*) are necessary for anthocyanin transport into the vesicles for storage [4]. Upstream of the structural and transporter genes, distinct transcriptional factors (TFs) are also involved. The most well-known MYB regulates the anthocyanin biosynthesis through an MYB-bHLH-WD40 (MBW) transcriptional factor complex, which directly activates key structural genes such as *DFR* and *UFGT*, operating at the final steps of the anthocyanin biosynthetic pathway [5, 6]. In the regulatory mechanism, basic helix-loop-helix (bHLH) TFs and tryptophan–aspartic acid (WD)-repeat proteins stabilize MYBs to form the MBW complex, which can enhance the expression activity of structural genes [7, 8]. Empirical evidence substantiates this basic model in apple, and different members such as *MdMYBA/1/10*, *MdMYB3*, *MdbHLH3*, *MdbHLH33*, and *MdTTG1* assemble into distinct MBW complexes to orchestrate anthocyanin accumulation [8–11]. Elucidation of these specific regulatory networks is meaningful for a comprehensive understanding of transcriptional regulation in anthocyanin biosynthesis and its integration.

5-Aminolevulinic acid (ALA), a naturally occurring δ-amino acid, is a pivotal precursor in the biosynthesis of all tetrapyrrole compounds, including chlorophyll and heme, and it also acts as a naturally occurring, environmentally friendly plant growth substance [12, 13]. One of the most outstanding regulatory effects of ALA on fruits is to promote apple anthocyanin accumulation [14]. This phenomenon has also been confirmed in litchi [15], peach [16], pear [17], and grapes [18]. Thus, ALA holds significant possibility for enhancing the production of high-quality fruits across various species. In apple, ALA enhances the gene expression related to anthocyanin biosynthesis such as *MdPAL*, *MdCHS* and *MdUFGT* [19, 20]. It can also promote gene expression involved in anthocyanin transport, such as *MdMATE8* and *MdGSTF12* [4, 21]. More important, ALA can upregulate many transcriptional factor genes expression, such as *MdMADS1* [22], *MdMYB9*/*10* [4], *MdERF78* [23], *MdHsfB3a* and *MdMYB110a* [24], where MdMYB110a, a paralog of MdMYB10 [25], transcriptionally upregulates *MdGSFT12* expression as well as anthocyanin accumulation. Recently, *MdNAC33* was verified to respond ALA-induction and directly bind to and activate the promoters of *MdDFR* and *MdANS* on the one hand and simultaneously form a transcriptional complex with *MdMYB1* on the other hand, to enhance the transcriptional activity of the latter on expression of *MdUFGT* and *MdGSTF12* [26]. In the synergistic module of MdWRKY71-MdMADS1, ALA-induced MdWRKY71 improves *MdMADS1* expression, then two TF proteins combine into two different forms of a TF complex. In the first form, MdWRKY71 is the master TF with MdMADS1 as an enhancing factor, directly binds to the W-boxes in the promoters of *MdANS* and *MdDFR*, regulates the gene transcription and anthocyanin biosynthesis. In another form, MdMADS1 targets the CArG-boxes in the promoters of *MdCHS* and *MdUFGT* with MdWRKY71 as an enhancing factor, enhancing the gene transcription and anthocyanin biosynthesis [27]. Furthermore, *MdFC2*, a ferrochelatase gene is involved in ALA-induced apple anthocyanin accumulation [28]. These findings suggest that ALA-induced apple anthocyanin accumulation undergo a highly complex regulatory mechanism, which needs further study.

B-box (BBX) proteins, a distinct subfamily of zinc-finger transcription factors, constitute a pivotal regulator of photomorphogenic development and diverse light-mediated physiological processes in plants [2]. These proteins are distinguished by the presence of either one or two evolutionarily conserved B-box zinc-binding motifs located at the N-terminal region and, in certain members, an additional CCT domain (CONSTANS, CO-like, and TOC1) positioned at the C-terminus. The modular configuration of these domains enables BBX proteins with the capacity to mediate precise transcriptional regulation in response to environmental cues. Functionally, BBX transcription factors participate in a broad spectrum of biological processes encompassing photoperiodic flowering, chloroplast development, hormonal signaling, and adaptive responses to both biotic and abiotic stressors [29]. The completion of plant genome sequencing has facilitated the identification of the BBX gene family, which includes 64 members in apple [30], 25 [31] or 37 in pear (*Pyrus bretschneideri*) [32], 32 in Arabidopsis [33], 30 in rice [34] or potato [35], 29 in tomato [36], and 24 in grapevine [37]. In apple, B-box (BBX) TFs play diverse roles in regulating anthocyanin biosynthesis through light-responsive signaling pathways. For example, MdBBX20 acts as a positive regulator, integrating UV-B and low temperature signals to promote pigment accumulation [38]. In contrast, MdBBX37 functions as a repressor by interacting with MdMYB1 and MdMYB9 to inhibit the binding to their target genes and, therefore, suppressing anthocyanin biosynthesis [39]. However, whether MdBBX TFs involvement in ALA-induced anthocyanin accumulation remain unreported.

In present study, we established that *MdBBX47* expression in apple was transcriptionally modulated by ALA, and exogenous ALA significantly enhanced *MdBBX47* promoter activity. Overexpressing (OE-)*MdBBX47* in apple fruits, leaves, and calli markedly enhanced anthocyanin accumulation, which was further strengthened by exogenous ALA. Conversely, RNA interfering (RNAi)-*MdBBX47* significantly suppressed the pigment formation. Molecular assay demonstrated that MdBBX47 directly bound to the promoters of *MdCHS* and *MdUFGT*, and furthermore, MdBBX47 can bind to the promoter of *MdMYB110a*, thereby promoting their transcriptional activity. Collectively, these findings identified MdBBX47 to be a key positive regulator in the ALA-induced anthocyanin biosynthesis pathway, facilitating better understanding of the regulatory mechanisms of ALA-induced anthocyanin accumulation and the improved application of ALA in high-quality fruit production.

## Materials and methods

### Plant materials and treatments

‘Orin’ calli, and ‘GL-3’ were granted by Professor Chunxiang You of Shandong Agricultural University for stable genetic transformation, which were cultured in the dark and sub-cultured every two weeks [40]. The ‘Huashuo’ fruits, harvested at the green mature stage in an orchard of Feng Xian County, Jiangsu Province, China, were utilized for both transient transformations. The other experimental materials, *Nicotiana benthamiana*, were already stored in the laboratory beforehand.

The bagged green mature apple (*Malus × domestica* cv. ‘Huashuo’) fruits were immersed in a 200 mg L⁻¹ ALA solution for 1 min, with distilled water serving as the control, and subsequently placed in the dark at room temperature overnight before exposure to light at 17 °C with an intensity of 200 µmol m⁻² s⁻¹ for 3 days. The apple peel samples were collected at 24, 48, and 72 h and stored at − 80 °C, with each treatment performed in triplicate as described by [24]. On the other hand, ‘GL-3’ apple plantlet leaves and ‘Orin’ apple calli were also used in the study, with plantlets maintained in a growth chamber at 24 °C under a photosynthetic photon flux density of 100 µmol m⁻² s⁻¹ on a 16-h light/8-h dark cycle and sub-cultured on MS medium monthly, whereas the ‘Orin’ calli were cultured under identical conditions but sub-cultured every two weeks, following the procedures described by [39].

When ALA was used to treat ‘GL-3’ mature apple leaves, the detached leaves from plantlets were laid flat on the supplemented MS medium with concentration of 0.25 mg L⁻¹ filter-sterilized ALA solution or without ALA in 9 cm diameter petri dishes, incubated overnight in darkness [24]. Then, petri dishes were moved to a light incubator set at 17 °C with a light intensity of 200 µmol m⁻² s⁻¹ to induce coloration. The samples were collected every 24 h over 72 h for subsequent analysis.

When ‘Orin’ calli were treated by ALA, the MS medium was prepared as described by [39], supplemented with 0.17 mg L⁻¹ ALA solution (filter-sterilized). The calli were sub-cultured on this medium in darkness for 12 h, followed by transfer to a light incubator at 17 °C with a light intensity of 200 µmol m⁻² s⁻¹ to induce coloration. The calli were collected every 7 days over 21 days for subsequent analysis.

### Analysis of anthocyanin content

After imaging. apple peel, leaves and callus tissue samples were subjected to extraction with 1% hydrochloric acid-methanol, and the homogenates were incubated in the dark at 4 °C for 24 h. The anthocyanin content was then determined by measuring absorbance at 530, 620, and 650 nm using a multifunctional microtiter plate reader (BioTek, Norcross, GA, USA), with results expressed as nanomoles of cyanidin-3-galactoside per gram of fresh tissue calculated by using a molar extinction coefficient of 3.43 × 10⁴, based on three biological replicates [4].

### Extraction of total RNA and real-time quantitative PCR (RT-qPCR) analysis

Total RNA was isolated from apple tissues using the Plant RNA Extraction Kit v1.6 (Biofit, Chengdu, China) according to the manufacturer’s protocol. Subsequently, cDNA synthesis was carried out using the One-Step gDNA Removal and cDNA synthesis supermix kit (TransGen Biotech, Beijing, China). The RT-qPCR was analyzed in a 20 µL reaction system containing 10 µL SYBR Green mix (with ROX), 0.4 µL 10 µmol L^− 1^ forward primer, 0.4 µL 10 µmol L^− 1^ reverse primer, 1 µL cDNA template (approximately 100 ng µL^− 1^), and 8.2 µL RNase-free water, using the QuantStudio 5 Real-Time PCR System (Applied Biosystems). The relative expression was calculated using the 2^−ΔΔCT^ method [41] and normalized by *MdActin*. All RT-qPCR primer sequences are detailed in Table S1.

### Assessment of MdBBX47 subcellular localization

The coding sequence (CDS) of *MdBBX47* (without the stop codon) was amplified and inserted into the pCAMBIA1302 vector. The empty vector (*35 S::GFP*) and the recombinant vector (*35 S::MdBBX47-GFP*) were transformed into *Agrobacterium tumefaciens* strain GV3101. Leaves from 4-week-old tobacco (*Nicotiana benthamiana*) plants expressing an mCherry-tagged nuclear marker were infiltrated with the *A. tumefaciens* suspension (OD_600_ = 0.6–0.8). In our study, the nuclear marker is actually NF-YA4-mCherry, which contains a nuclear localization signal (NLS) via fusion to the nuclear transcription factor NF-YA4. The infiltrated tobaccos were then kept in darkness for 12 h, then transferred to a plant growth chamber for 48–60 h. Confocal microscopy (LSM-800, Zeiss) was used to detect the GFP and mCherry fluorescence signals. The primers are listed in Table S1.

### Bioinformatics characterization, phylogenetic analysis and multiple sequence alignment

Protein sequences used for phylogenetic analysis were retrieved from the National Centre for Biotechnology Information (https://www.ncbi.nlm.nih.gov/) and Genome Database for Rosaceae (https://www.rosaceae.org/) database. Conserved motifs were projected using MEME Suite (https://meme-suite.org) with a maximum number of 10 and functional domains were annotated using NCBI Batch-CD Search (https://www.ncbi.nlm.nih.gov/Structure/bwrpsb/bwrpsb.cgi). A phylogenetic tree was constructed using the neighbor-joining method in MEGA 11.0.10 (https://www.megasoftware.net/) to analyze the evolutionary relationships between MdBBX47 and homologous BBX proteins from other species involved in anthocyanin biosynthesis. PlantCARE (http://bioinformatics.psb.ugent.be/webtools/plantcare/html/) was used to analyze *cis*-elements in the promoter region. All sequences were aligned using DNAMAN for multiple sequence comparison. Protein sequences of these homologous BBX members are listed in Table S2.

### Activation of ALA on MdBBX47 promoter activity

To investigate the influence of ALA on the promoter activity, a 2,000 bp fragment of the *MdBBX47* promoter located upstream of the start codon was cloned into the pBI121-GUS vector to generate the *ProMdBBX47*-GUS construct, this construct was introduced into the *A. tumefaciens* GV3101 and transiently transformed apple leaves, followed by treatment with 0.5 mg L⁻¹ ALA and subsequent GUS staining was performed according to [26]. Primer sequences are listed in Table S1.

### Transformation of MdBBX47 into apple calli, leaves, and fruits

To generate the overexpression vector, the coding sequence (CDS) of *MdBBX47* was cloned into the pCAMBIA1302 vector to generate an overexpression construct (OE-*MdBBX47*). At the same time, a specific fragment of the *MdBBX47* CDS was inserted into the pHELLSGATE4 vector via Gateway BP to generate an RNA interference construct (RNAi-*MdBBX47*). Primer sequences used are listed in Table S1. The resultant recombinant vectors were introduced into *A. tumefaciens* strains LBA4404 or EHA105. LBA4404 was used to infect ‘Orin’ apple calli to establish stably transgenic lines, as previously described [23], whereas, EHA105 was used for transient transformation of ‘GL-3’ leaves and “Huashuo” fruits, following the method of [26].

For leaf infection, detached leaves were immersed in the EHA105 bacterial suspension carrying the recombinant vector and subjected to vacuum infiltration for 10 min. The leaves were then incubated overnight in darkness, followed by 3 days of continuous illumination in a growth chamber. Subsequently, the leaves were documented in photos, and samples were collected for anthocyanin content measurement and RNA extraction.

For fruit infection, the apple peels were injected with the EHA105 bacterial suspension. After 24 h in darkness, the fruits were exposed to continuous light at 200 µmol m⁻² s⁻¹ for 3 days. The injected areas were then documented in photos, and samples were collected for anthocyanin quantification and expression of gene analysis as described above. Primer sequences are listed in Table S1.

### Yeast one-hybrid (Y1H) assays

The Y1H assay was performed to verify the binding of MdBBX47 to the promoters of downstream target genes. A 2,000 bp fragment promoter upstream of start codon of *MdCHS*, *MdF3H*, *MdDFR*, *MdANS*, *MdUFGT* and *MdMYB110a* were individually cloned into the pHIS2 vector served as the bait plasmid, while the full-length coding sequence CDS of *MdBBX47* was cloned into the pGADT7(AD) vector to generate AD-MdBBX47 prey vector, using the empty AD vector as the negative control. To detect and suppress the self-activation of the *ProMdCHS-pHIS2*, *ProMdUFGT*-*pHIS2*, and *ProMdMYB110a-pHIS2* plasmid, the prey vector (AD-MdBBX47) was co-transformed with *ProMdCHS-pHIS2*, *ProMdUFGT-pHIS2*, and *ProMdMYB110a-pHIS2* into Y187 on the double dropout (DDO) medium (SD/−Trp − Leu) and triple dropout (TDO) medium (SD/−Trp − Leu−His) containing 75 mM 3-AT for 3 d in a 29 °C incubator. The bacterial colony growth was then photographed. Primers for vector construction are listed in Table S1.

### Luciferase reporter gene assay (LUC)

The full-length CDS of *MdBBX47* was recombined into the pCAMBIA1302 vector to generate the *35 S::MdBBX47* effector vector, which was individually transformed into *A. tumefaciens* GV3101. The promoter sequences of *MdCHS*, *MdUFGT*, and *MdMYB110a* were individually cloned into pGreenII0800-LUC to construct the reporter vectors, which were then transformed into *Agrobacterium tumefaciens* GV3101 (pSoup), and various combinations of reporters and effectors were subsequently infiltrated into 4-week-old tobacco leaves. After a 36-h period in darkness, the LUC signal was monitored using a living imaging apparatus (PIXIS 1024 B, Princeton, United States). The relative fluorescence intensity was analyzed using ImageJ software. Primers for vector construction are listed in Table S1.

### Electrophoretic mobility shift assay (EMSA)

The *MdBBX47* CDS was cloned separately into the pET32a (6×His) vector. Then, the fusion protein MdBBX47-His was expressed in *Escherichia coli* BL21, which was purified using HIS purification columns of a commercial kit (Cwbiotech, Beijing, China). Biotin-labeled EMSA probes were synthesized, and EMSA was conducted by following the manufacturer’s instructions (LightShift Chemiluminescent EMSA Kit; Thermo Fisher Scientific, Shanghai, China). Primers for vector construction are listed in Table S1.

### Histochemical GUS staining analysis

The 2,000 bp upstream promoter regions of *MdCHS*,* MdUFGT* and *MdMYB110a* were fused with the pBI121-GUS plasmid to replace the CaMV35S promoter as the reporter, while the full-length CDS of *MdBBX47* was inserted into a CaMV35S-driven vector as the effector, with primers listed in Table S1. Briefly, plant materials were incubated in GUS staining buffer (1 mM X-Gluc, 0.5 mM ferrocyanide, 0.5 mM ferricyanide, 0.1% Triton X-100, and 0.1 mM EDTA) for staining, followed by destaining with absolute ethanol. The generated constructs were transformed into the Agrobacterium strain GV3101 and then infiltrated into tobacco leaves. GUS staining was performed on the leaves after 3 days.

### Yeast two-hybrid (Y2H) assays

In order to verify whether MdHY5 interacts with MdBBX47, Y2H assays of two proteins were performed. The CDS of *MdHY5* was cloned into the pGBKT7 vector (serving as the bait) and the CDS of *MdBBX47* into the pGADT7 vector (serving as the prey). Bait-prey plasmid pairs were co-transformed into Y2HGold chemically competent cell (Coolaber, Beijing, China) via lithium acetate transformation. AD empty vector with bait plasmid or BD empty vector with prey plasmid was used the negative controls. Transformed yeast were first cultured on SD/−Trp/−Leu (DDO) medium at 30 °C for 3–5 days. Positive colonies were then plated on SD/−Ade/−His/−Trp/−Leu (QDO) medium supplemented with X-α-Gal (20 mg mL^− 1^) to assess protein–protein interactions. Interaction was confirmed based on colony growth and development of blue color resulting from α-galactosidase activity [42].

### Luciferase complementation imaging assays (LCI)

The CDS of *MdBXB47* without the stop codon was ligated to the pC1300-cLUC vector. Meanwhile, the CDS of *MdHY5* with its stop codon was ligated to the pC1300-nLUC vector. Constructs were transformed into *Agrobacterium tumefaciens* GV3101 (pSoup-p19), and the resulting transformed *A. tumefaciens* suspensions were infiltrated into *N. benthamiana* leaves. After 3 days, LUC signals were imaged using a CCD camera (PIXIS 1024B, Princeton, USA) [43].

### Statistical analysis

All data are expressed as the means of at least three independent biological replicates. Statistical analysis was conducted using Student’s *t*-test, one-way or two-way ANOVA, followed by Tukey’s HSD test or Duncan’s multiple range test for mean separation.

## Results

### ALA promotes apple coloration and anthocyanin accumulation in different tissues

**Fig. 1:**
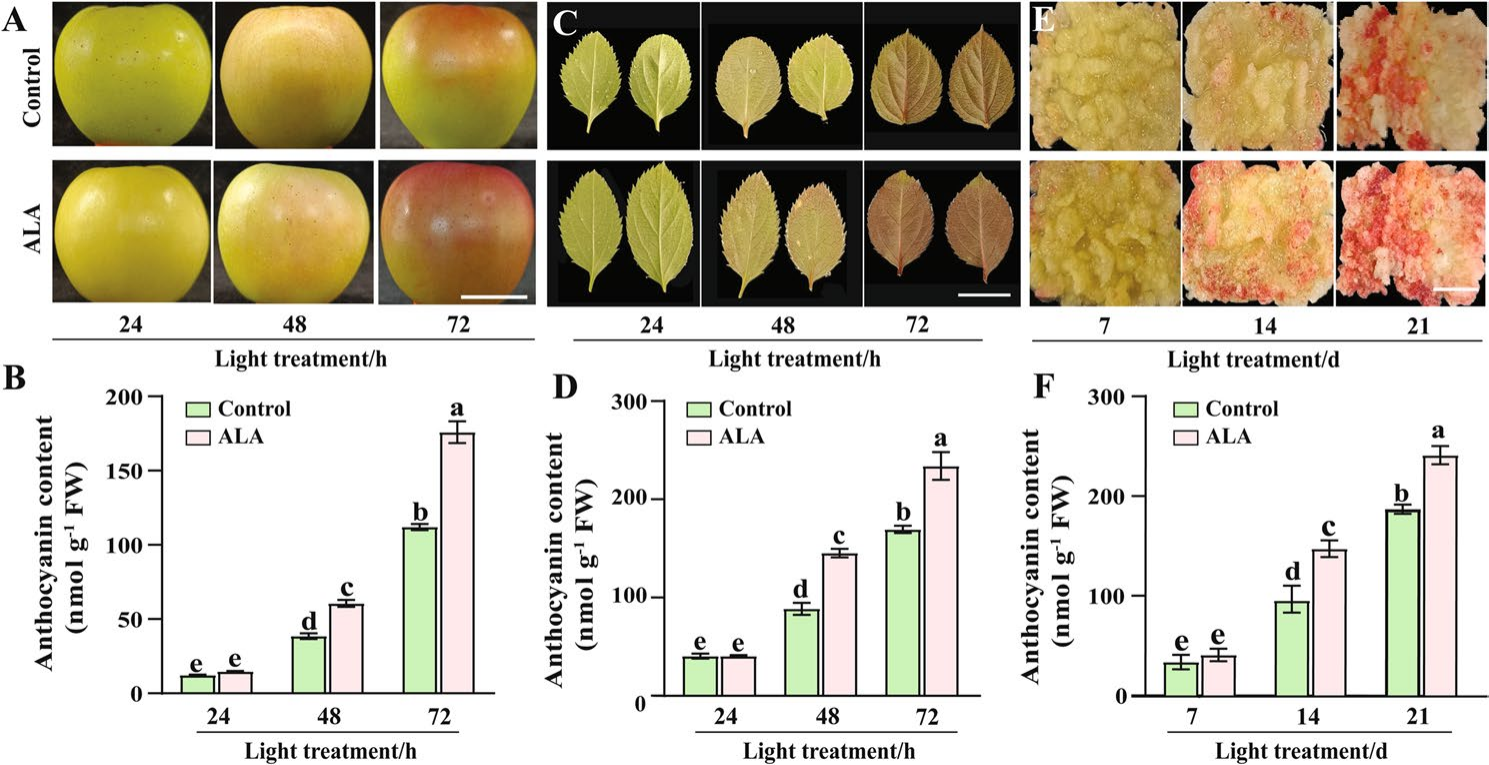
ALA promotes anthocyanin accumulation in different apple tissues. **A** For color development, apple fruits were immersed in a 200 mg L⁻¹ ALA solution for 1 minute, with distilled water serving as the control, after 12 h of ALA treatment in darkness followed by 72 h under light (200 µmol m⁻² s⁻¹) at 17°C. Scale bar = 5 cm. **B** Anthocyanin content in apple fruits after continuous light exposure. **C** Coloring of apple leaves treated with 0.25 mg L^− 1^ ALA for 12 h in darkness and then 72 h light exposure (200 µmol m⁻² s⁻¹) at 17°C. Scale bar = 1 cm. **D** Anthocyanin content in apple leaves. **E** The ‘Orin’ apple calli were treated with 0.17 mg L⁻¹ ALA for 12 h in darkness, followed by 21 days under light (200 µmol m⁻² s⁻¹) at 17 °C, resulting in coloration. Scale bar = 1 cm. **F** Anthocyanin content in the ‘Orin’ calli. Data are presented as means ± SE of three biological replicates, with different letters in each panel indicating significant differences (two-way ANOVA, *P <* 0.05)

It is well established that ALA induces anthocyanin accumulation in apple fruits and cultured calli [44]. In the present study, we treated apple fruits, cultured calli and detached leaves with exogenous ALA, and found that ALA can enhance anthocyanin accumulation in various tissues of apples (Fig. 1). From Fig. 1A and B, the apple peels turned redder than the control after 24 h of light exposure, however, the anthocyanin content was not significantly different. When fruits were light-exposed for 48 h, the anthocyanin content in ALA-treated samples increased by approximately 1.5-fold compared with the control which was further increased to nearly two-fold after 72 h of illumination (Fig. 1B). Similarly, ALA significantly improved enhancement in pigmentation in the detached leaves (Fig. 1C, D) and calli (Fig. 1E, F). These results suggest that exogenous ALA can improve anthocyanin accumulation in different tissues of apple.

### MdBBX47 expression is upregulated by ALA during apple coloration

**Fig. 2:**
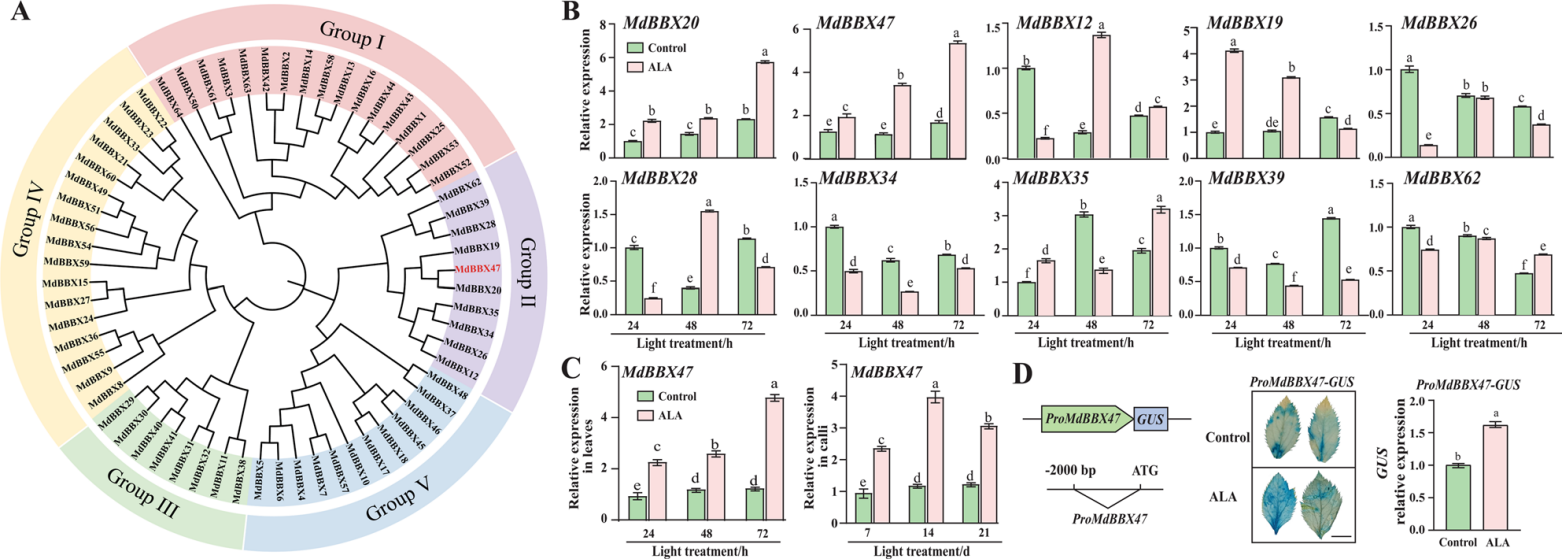
Screening of MdBBX47 as an ALA responsive factor during ALA-induced anthocyanin accumulation in apples. **A** The phylogenetic tree using MEGA 11.0.10 was constructed by the neighbor-joining method with 1000 bootstrap replicates of *MdBBX* family members in apple genome. Sixty-four *MdBBX* members can be divided into five groups, named as group I–V. Among them, *MdBBX20* and *MdBBX47* are very close, and fall in Group II. Since *MdBBX47* is the main gene investigated in this study, we specifically highlight it in red font. **B** The expression profiles of the *MdBBX* members of Group II in ALA-treated apple peels, as described in Fig. 1A. **C** The *MdBBBX47* expression in the detached leaves and calli of apple after ALA treatment, which was described in Fig. 1C and E, respectively. **D** GUS staining assays demonstrated that the promoter of *MdBBX47* is activated by exogenous ALA treatment. Recombinant gene construct is listed in the left, GUS staining of *ProMdBBX47-GUS* was performed in the transgenic apple leaves, which were treated with 0.25 mg L⁻¹ ALA or left untreated. On the right is the relative expression of the GUS gene. Data are presented as means ± SE of three biological replicates, with different letters in each panel indicating significant differences (two-way ANOVA, *P <* 0.05 used for B, C and Student’s *t-*test, *P* < 0.05 applied for D)

A comprehensive genome-wide analysis of the MdBBX family in the apple genome showed that 64 members can be divided into five distinct groups (Fig. 2A). Among them, 17 *MdBBX* genes are in Group I, 10 in Group II, 8 in Group III, 17 in Group IV, and 12 in Group V. MdBBX20, a member of Group II, has been reported to positively regulate apple anthocyanin accumulation; therefore, we focused on other members of Group II [38].

However, we analyzed the gene structure, conserved motifs, and domains of 10 MdBBX members of Group II (Fig. S2), detected ten members of *MdBBX* in Group II by RT-qPCR for their expression in apple peels after ALA treatment. The results indicated that the expression of *MdBBX20* and *MdBBX47* was significantly upregulated by ALA, while the others, including *MdBBX12*/*19*/*26*/*28*/*34*/*35*/*39*/*62* had no such stable responses (Fig. 2B). Comparatively, *MdBBX47* was more responsive to ALA than *MdBBX20*. In the detached leaves and the calli, we also observed that exogenous ALA significantly promoted *MdBBX47* expression (Fig. 2C). These verified that MdBBX47 is a suitable gene for further study.

In order to test the promotion of ALA on the transcriptional activity of *MdBBX47* promoter, we performed GUS staining and gene expression assay. The results showed that ALA treatment stimulated the transcriptional activity of the promoter of *MdBBX47* (Fig. 2D). These findings indicate that *MdBBX47* is an ALA-responsive factor, which may be contributed in ALA-induced anthocyanin accumulation in apple.

### Conservation analysis and subcellular localization of MdBBX47

**Fig. 3:**
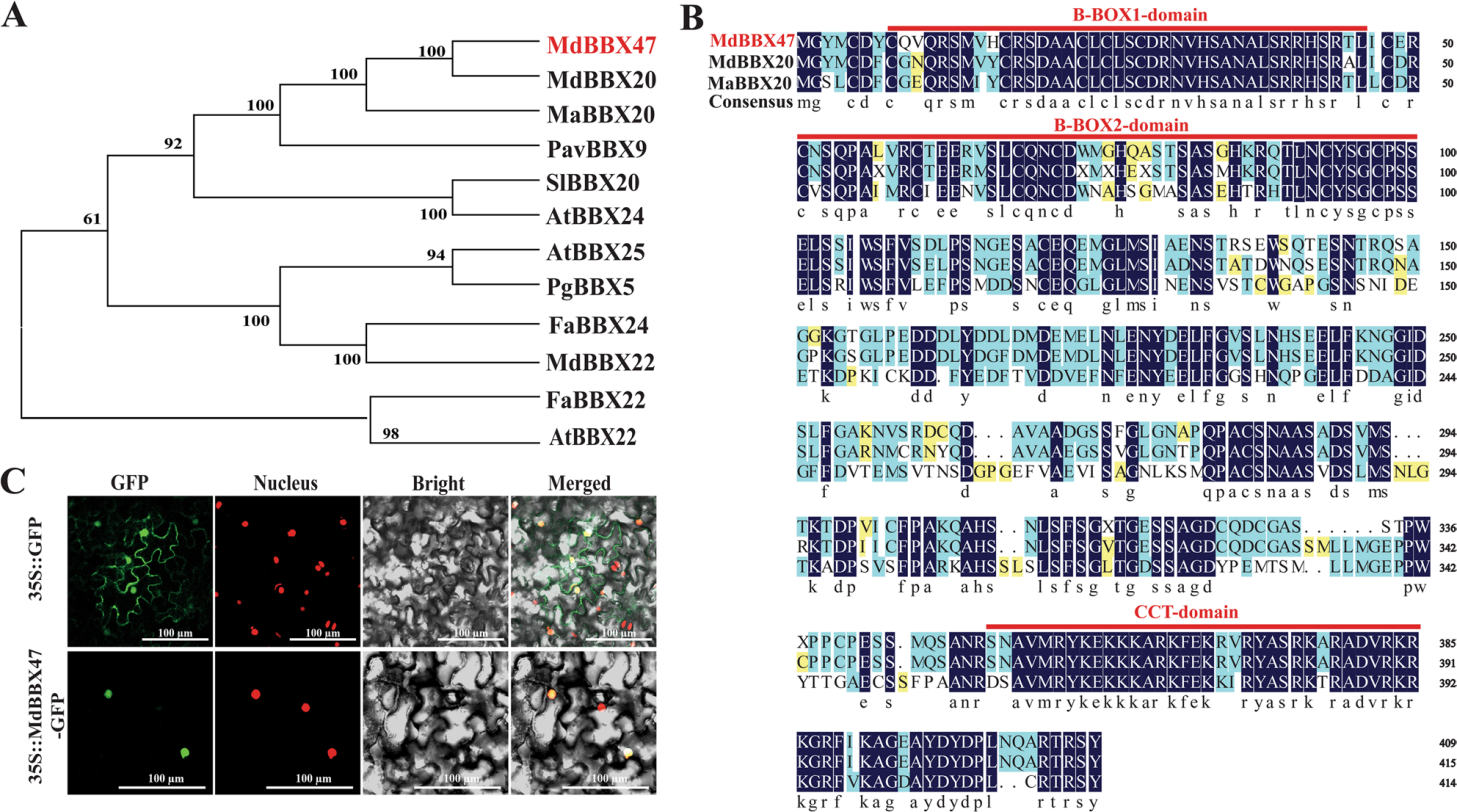
Conservation analysis of MdBBX47 and subcellular localization. **A** Phylogenetic tree was constructed using MEGA 11.0.10 and the neighbor-joining method with 1000 bootstrap replicates of MdBBX47 with the homologs from various species involved in anthocyanin regulation, including apple (*Malus domestica*), tomato (*Solanum lycopersicum*), Arabidopsis (*Arabidopsis thaliana*), strawberry (*Fragaria ananassa*), sweet cherry (*Prunus avium*), grape hyacinth (*Muscari aucheri*), and pomegranate (*Punica granatum* L.). **B** Amino acid sequence alignments were generated using DNAMAN software of apple MdBBX47 with homologs. Two highly conserved domains of the B-BOX protein and a CCT-domain are highlighted with red lines. The black color indicates 100% similarity, blue indicates 75% and yellow indicates 50% similarity, respectively. The consensus amino acid sequence is provided below the alignments. **C** Subcellular localization observation using fluorescence microscopy of MdBBX47. The constructs of *35 S::GFP* served as control and NF-YA4-mCherry served as a nuclear marker, *35 S::MdBBX47-GFP* were transiently expressed in tobacco leaves, and confocal microscopy demonstrated that MdBBX47 is localized in the nucleus. Scale bar = 50 μm

To better understand the characteristics of MdBBX47, we constructed a phylogenetic evolutionary tree of BBXs across various species including AtBBX22 [44, 45], AtBBX24, and AtBBX25 [46] in Arabidopsis, MdBBX20 [38] and MdBBX22 [47] in apple, PgBBX5 [48] in pomegranate, PavBBX9 in sweet cherry [49], SlBBX20 in tomato [50], MaBBX20 in grape hyacinth [51], FaBBX22 and FaBBX24 in strawberry [52, 53]. All of the BBX TFs were reported involved in the regulation of anthocyanin biosynthesis (Fig. 3A). Comparatively, MdBBX47 and MdBBX20 are very close, sharing with an identity of 86.99%. MaBBX20 is also close to MdBBX20 and MdBBX47, with an estimated overall sequence similarity of 77.99%, supporting their grouping in the same clade. Amino acid sequence alignment showed that the mainly conservative regions are the B-BOX domains and CCT-domain, while the rest of amino acid sequences is rather divergent (Fig. 3B).

To elucidate subcellular localization of MdBBX47, a *35 S::MdBBX47-GFP* fusion construct was transiently expressed in *Nicotiana benthamiana* epidermal cells, with *35 S::GFP* serving as the control. Observation using fluorescence microscopy 60 h post infiltration showed that an exclusive GFP fluorescence signal was detected in the nuclei, matching with the localization of the nuclear marker protein (NF-YA4-mCherry), while the fluorescent signals from the empty plasmid were observed in both the nucleus and cytosol (Fig. 3C). These findings conclusively establish that MdBBX47 is a nucleus localized protein, consistent with its predicted role as a transcription factor.

### MdBBX47 is necessary in ALA-induced apple anthocyanin accumulation

**Fig. 4:**
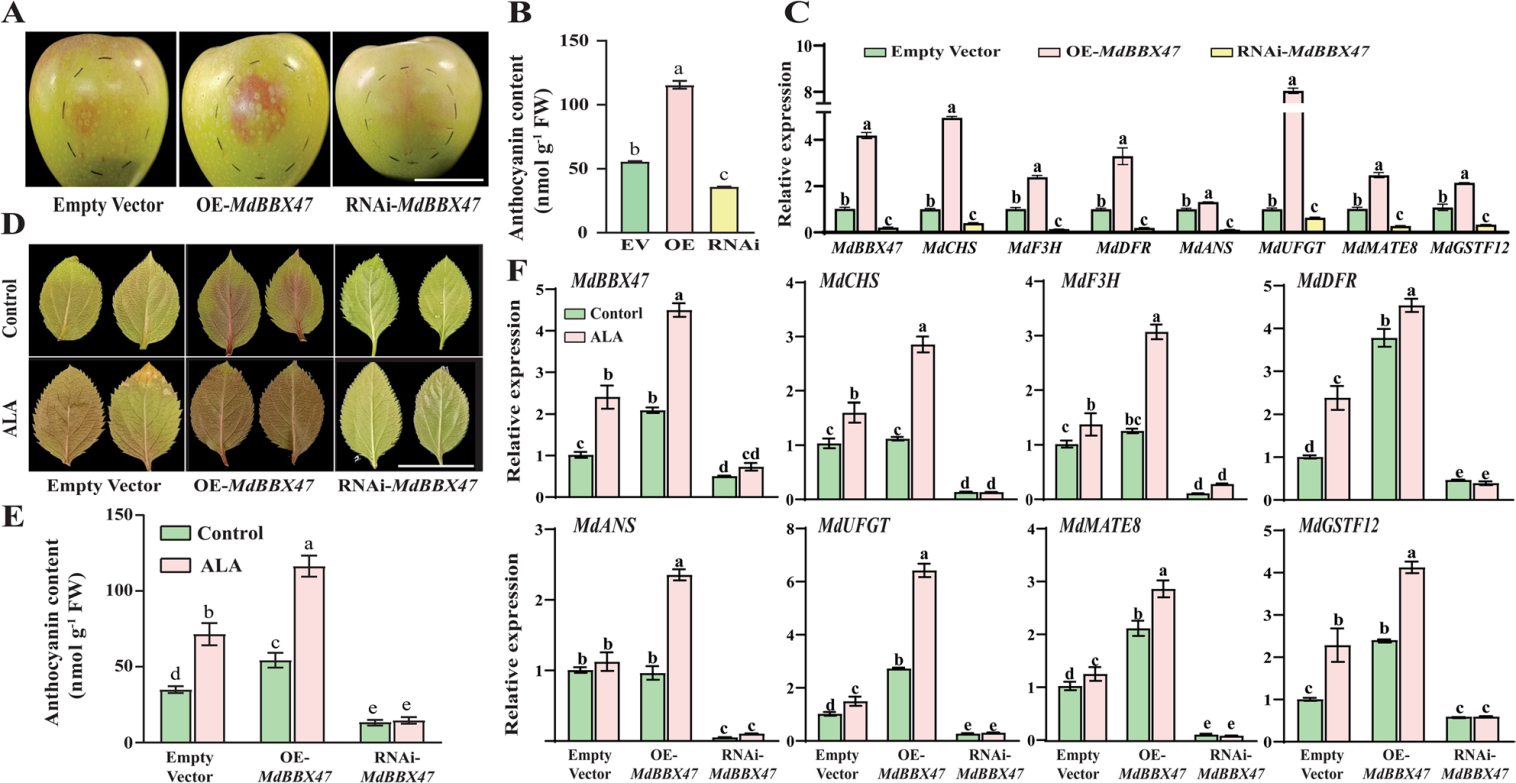
MdBBX47 mediates ALA-induced anthocyanin accumulation in apples. **A** Color development in apple fruits injected with OE-*MdBBX47* or RNAi-*MdBBX47* vectors, with an empty vector (pCAMBIA1302) as the control. Black circles indicate the injection sites. Scale bar = 5 cm. **B** Anthocyanin content in the injected fruit skins. **C** Expression pattern of *MdBBX47* and structural genes related to anthocyanin biosynthesis and transport. **D** Appearance of apple leaves treated with 0.25 mg L⁻¹ ALA. EV: empty vector; OE-*MdBBX47*: overexpression of *MdBBX47*; RNAi-*MdBBX47*: RNA interference of *MdBBX47*. Scale bar = 5 mm. **E** Anthocyanin content in transiently transformed apple leaves. **F** Relative expression of *MdBBX47* and the genes related to anthocyanin biosynthesis and transport in apple leaves treated by ALA. Data are presented as means ± SE of three biological replicates, with different letters within each panel indicating statistically significant differences (**B** and **C** were used for one way while **E** and **F** were used for two-way ANOVA, *P <* 0.05)

To elucidate the function of *MdBBX47* in the regulation of anthocyanin accumulation in apples, transiently overexpressing (OE)-*MdBBX47* and RNA interfering (RNAi)-*MdBBX47* constructs were introduced via *Agrobacterium tumefaciens*-mediated transformation into apple peels or leaves. In the fruit peels, OE-*MdBBX47* markedly enhanced red pigmentation, whereas RNAi-*MdBBX47* resulted in a visible reduction in coloration relative to the empty vector (Fig. 4A). Anthocyanin quantification demonstrated that OE-*MdBBX47* promoted anthocyanin accumulation, whereas RNAi-*MdBBX47* suppressed anthocyanin synthesis (Fig. 4B). Furthermore, we examined the expression levels of *MdBBX47* and key anthocyanin biosynthetic structural and transport genes in transiently transformed fruits. In the peel of apple fruits, OE-*MdBBX47* upregulated expression of *MdBBX47* along with significant activation of *MdCHS*,* MdF3H*, *MdDFR*, *MdANS*, *MdUFGT*, *MdGSTF12*, and *MdMATE8*. Conversely, the gene expression was all significantly repressed in RNAi-*MdBBX47* peels (Fig. 4C). These suggest that *MdBBX47* functions upstream of the anthocyanin synthesis and transport genes.

To substantiate the regulatory role of *MdBBX47* in anthocyanin biosynthesis, different vectors were introduced in the apple leaves. It was found that OE-*MdBBX47* was beneficial to anthocyanin biosynthesis, while suppression of *MdBBX47* hindered anthocyanin accumulation. In addition, exogenous ALA enhanced anthocyanin accumulation in both EV and OE-*MdBBX47* leaves, but the improvement was eliminated in RNAi-*MdBBX47* (Fig. 4D, E). These results indicate that MdBBX47 plays a crucial role in the accumulation of anthocyanin in apple leaves induced by ALA. Furthermore, OE-*MdBBX47* promoted the gene expression including *MdBBX47* along with *MdCHS*, *MdF3H*, *MdDFR*, *MdANS*, *MdUFGT*, *MdMATE8*, and *MdGSTF12*, with ALA further promoted their gene expression, whereas RNAi-*MdBBX47* inhibited the gene expression, and ALA had no rescue effect (Fig. 4F). These results collectively establish MdBBX47 as a positive regulator in ALA-induced anthocyanin accumulation in apple fruits and leaves.

### MdBBX47 alters the expression of genes related to anthocyanin accumulation in apple calli

**Fig. 5:**
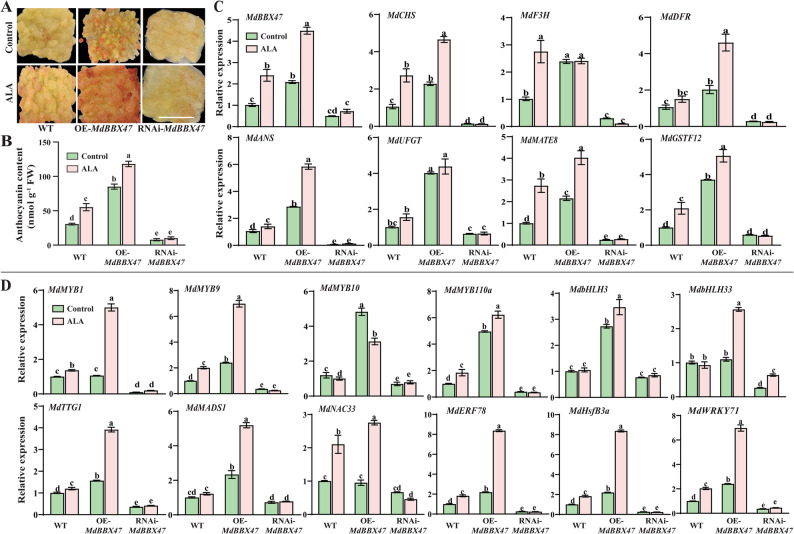
Effect of *MdBBX47* on the expression of anthocyanin biosynthetic and transport-related genes as well as the relevant transcription factors in ALA-treated transgenic apple calli. **A** Coloration comparison of ‘Orin’ apple calli, transformed with OE-*MdBBX47* or RNAi-*MdBBX47* vectors, and cultured on MS media containing 0.17 mg L^− 1^ ALA, Scale bar = 2 cm. **B** Anthocyanin content in different genotypic apple calli. **C** Relative expression levels of *MdBBX47* in the transgenic apple calli and relative expression levels of the genes related with anthocyanin biosynthesis and transport in ALA-treated apple calli. **D** Relative expression of the transcription factor genes previously identified as a role in ALA-induced anthocyanin accumulation, the data are presented as means ± SE from three biological replicates, with different letters in each panel denoting significant differences (two-way ANOVA, *P* < 0.05)

In order to evaluate the assessment of *MdBBX47* in response to ALA treatment, we generated stable transgenic *OE-MdBBX47* and *RNAi-MdBBX47* apple calli, which were validated through PCR and RT-qPCR. (Fig. S1) and cultured on MS media with 0.17 mg L^− 1^ ALA or not [27]. After two weeks light culture, OE-*MdBBX47* significantly increased anthocyanin content, while RNAi-*MdBBX47* led to a marked reduction in anthocyanin accumulation (Fig. 5A, B). Furthermore, exogenous ALA promoted anthocyanin accumulation in the both WT and OE-*MdBBX47* calli, but not in RNAi-MdBBX47, suggesting that MdBBX47 mediation is necessary for ALA-induced anthocyanin accumulation.

We assessed the relative expression of genes associated with anthocyanin biosynthesis and transported in ALA-induced apple calli. As shown in (Fig. 5C), all examined genes including *MdBBX47*, *MdCHS*, *MdF3H*, *MdDFR*, *MdANS*, *MdUFGT*, *MdMATE8*, and *MdGSTF12* were upregulated in OE-*MdBBX47* lines. In contrast, RNAi-*MdBBX47* led to the downregulation. ALA improved gene expression in the OE lines, but not in RNAi. These results suggest that MdBBX47 plays a crucial role in ALA-induced anthocyanin accumulation in apple calli.

To investigate potential effects of *MdBBX47* on transcription factor gene expression, we conducted a comprehensive RT-qPCR analysis to examine the expression of twelve key transcription factor genes, that have been recently reported involved in ALA-induced anthocyanin accumulation. As shown in (Fig. 5D), most of the genes including *MdMYB9*/*10*/*110a*, *MdbHLH3*, *MdTTG1*, *MdMADS1*, *MdERF78*, *MdHsfB3a*, and *MdWRKY71* were consistently upregulated in OE*-MdBBX47* calli and downregulated in *RNAi-MdBBX47*. However, the expression of *MdMYB1*, *MdbHLH33*, and *MdNAC33* was not upregulated by OE-*MdBBX47*, although the expression levels were reduced by RNAi-*MdBBX47*. Furthermore, exogenous ALA significantly enhanced most of TF gene expression, including *MdMYB1*/*9*/*110a*, *MdTTG1*, *MdNAC33*, *MdEFR78*, *MdHsfB3a* and *MdWRKY71*, but the improvement was eliminated by RNAi-*MdBBX47*. Since *MdMYB1*/*9*/*10*/*110a* and *MdTTG1* belong to the MBW complex components, while *MdNAC33*, *MdHsfB3a*, *MdMADS1*, and *MdWRKY71* regulate anthocyanin accumulation in non-MBW routes, the findings here reinforce the hypothesis that ALA-induced MdBBX47 acts as an upstream regulator within both MBW and non-MBW transcriptional activation cascades to promote anthocyanin accumulation.

### MdBBX47 activates transcription of MdCHS and MdUFGT by directly binding to their promoters

**Fig. 6:**
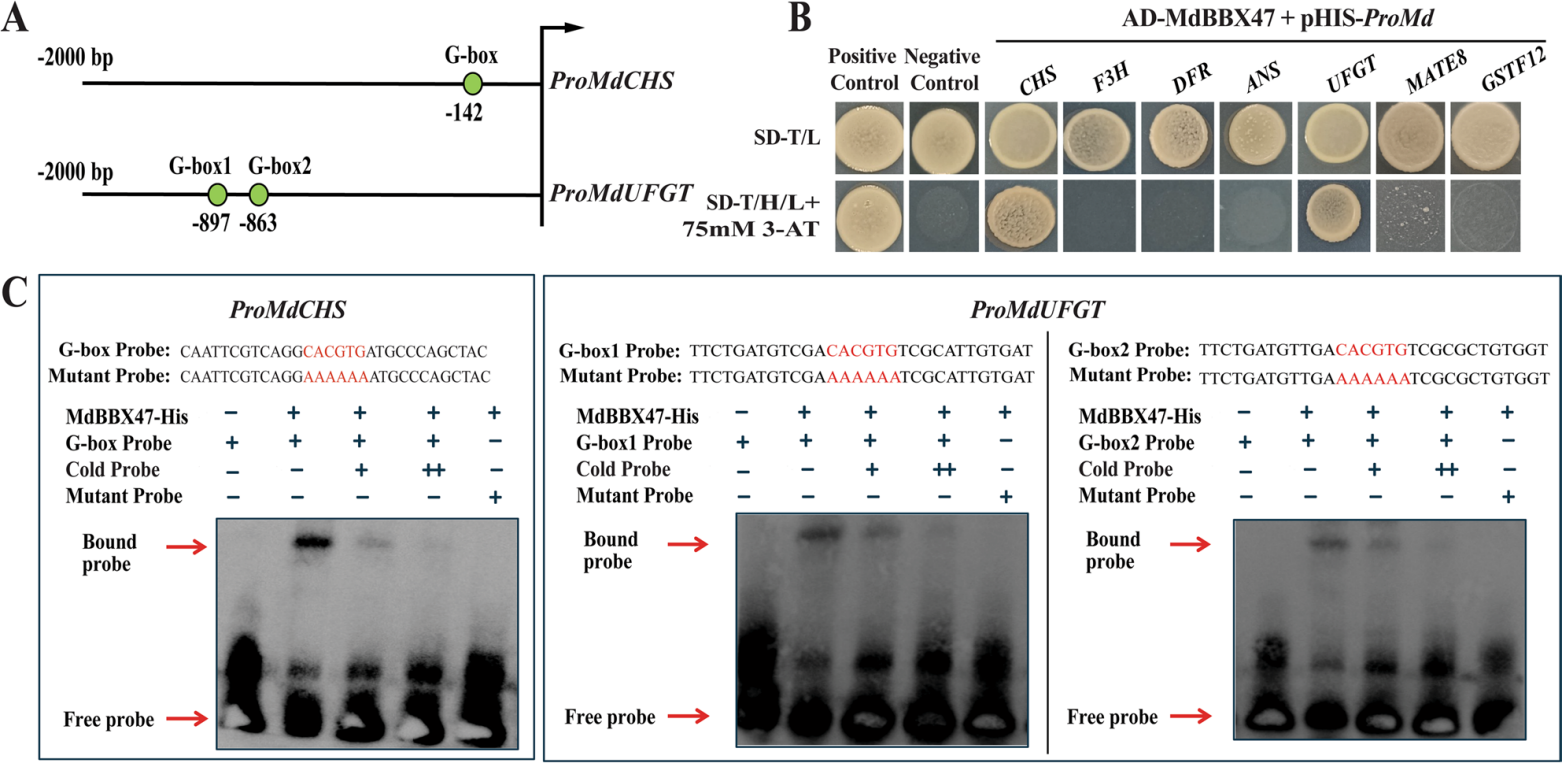
Validation of MdBBX47 binding to promoters of key anthocyanin-related genes. **A** Distribution of G-box *cis*-elements in *MdCHS* and *MdUFGT* promoters. **B** Y1H analysis of the possible interaction of MdBBX47 with the promoters of *MdCHS*,* MdF3H*,* MdDFR*,* MdANS*,* MdUFGT*,* MdMATE8*, and *MdGSTF12*, which are responsible for anthocyanin biosynthesis and transport. The pGADT7-Rec2-53 along with pHIS2-p53 acted as the positive control, while the pGADT7 and pHIS2 empty-vector mixture served as the negative control. SD/−T/−L is synthetic dropout medium lacking tryptophan and leucine, while SD/−T/−H/−L represents synthetic dropout medium lacking leucine, tryptophan, and histidine, with 75 mM 3-AT. **C** EMSA shows specifically direct binding of MdBBX47-His fusion protein to the *cis*-acting elements (CACGTG) of the *MdCHS* and *MdUFGT* promoters. The cold probe represents an unlabeled competitive probe. The ‘+’ stands for presence and the ‘–’ stands for absence. Cold probes are designated as ‘++’

To identify the interaction ability of MdBBX47 with the target gene promoters, we first analyzed the *cis*-elements (G-box, CACGTG) in the promoters of all genes related to anthocyanin biosynthesis and transport, however, only in the *MdCHS* and *MdUFGT* promoters we found one or two the motifs (Fig. 6A). Then, we cloned 2,000 bp promoter regions of the structural genes to conduct yeast one-hybrid (Y1H) assays. The empty vector AD was not used as a control in the Y1H assay because our focus was on specifically testing the binding activity of *MdBBX47* with the target gene promoters. The results demonstrated that only yeast co-transformed with AD-*MdBBX47* and *ProMdCHS-pHIS2* or *ProMdUFGT-pHIS2* that can grow normally on SD/−Trp/−His/−Leu media supplemented with 75 mM 3-AT (Fig. 6B). These confirm that MdBBX47 can bind to the promoters of *MdCHS* and *MdUFGT*, but not to the others, including *MdF3H*, *MdDFR*, *MdANS*,* MdMATE8* and *MdGSTF12*. Then, we performed EMSA to evaluate the specificity of MdBBX47 binding to the G-box motifs of *MdCHS* and *MdUFGT* promoters. Biotin-labelled promoter fragments were utilized as probes in the EMSA, revealing that MdBBX47 can bind to the G-box motifs in both promoters, but decreased with increasing concentrations of unlabeled (cold) probes. No binding was detected when the mutant probe was added (Fig. 6C). These confirm that MdBBX47 can specifically bind to the G-box elements of *MdCHS* and *MdUFGT* promoters.

### Verification of MdBBX47 activating the promoters of MdCHS and MdUFGT promoters

**Fig. 7:**
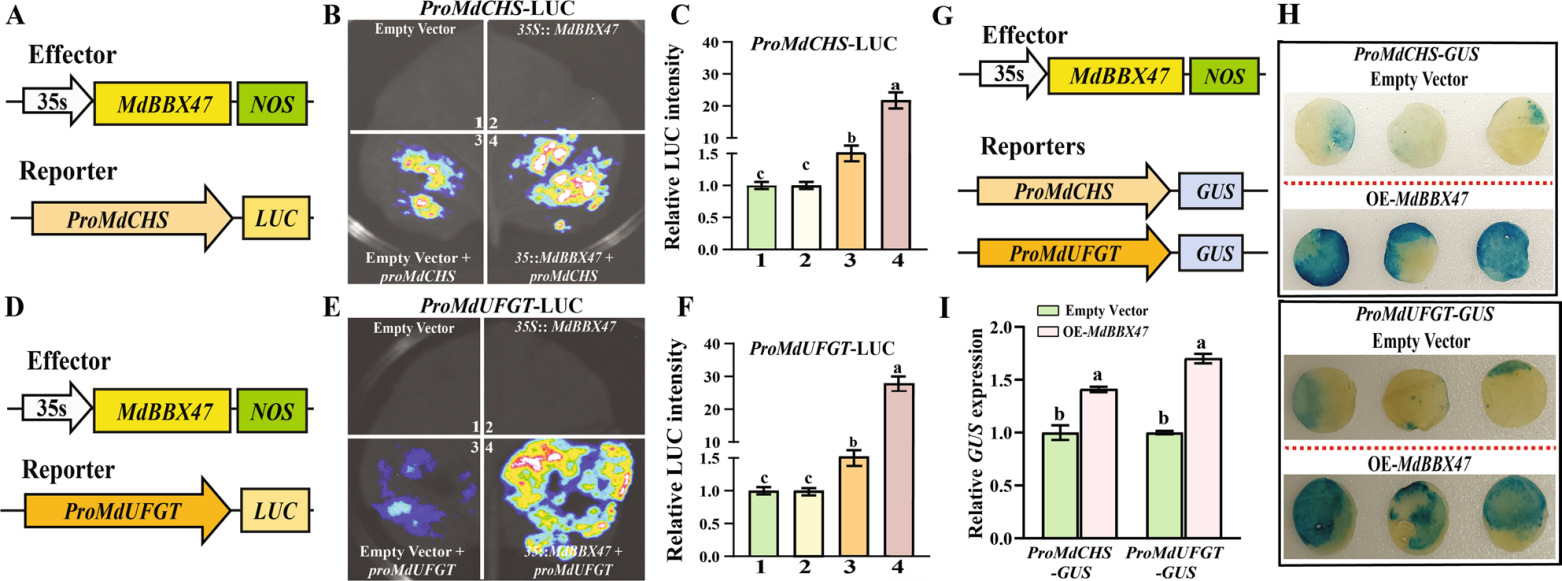
Transcriptional activation of MdBBX47 on the promoters of *MdCHS* and *MdUFGT*. **A**, **D** Schematic diagrams of LUC analysis plasmid construction. **B** Luminescence observations in tobacco leaves. 1: Empty Vector + *LUC*, 2: *35 S::MdBBX47 + LUC*, 3: Empty vector + *ProMdCHS*, 4: *35 S::MdBBX47* + *ProMdCHS.* (**E**) 1: Empty Vector + *LUC*, 2: *35 S::MdBBX47* + LUC, 3: Empty vector + *ProMdUFGT.* 4: *35 S::MdBBX47* + *ProMdUFGT*. **C**, **F** Relative LUC luminescence intensity. The data represent the means of three biological replicates, and different letters above bars in a panel indicate significant differences (*P* < 0.05). **G** Schematic diagrams of the effector and reporter vectors used in the *GUS* assays. **H** GUS staining of tobacco leaves demonstrated that MdBBX47 activates the promoters of *MdCHS* and *MdUFGT*. **I** Quantitative analysis of *GUS* expression in tobacco leaves. Data are presented as means ± SE of three biological replicates, with different letters indicating significant differences (*P <* 0.05, Student’s *t-*test)

To validate the transcriptional activation of MdBBX47, we conducted LUC and GUS assays to assess the function of MdBBX47 on the downstream target genes. The 2,000 bp promoter regions of *MdCHS* and *MdUFGT* were cloned and inserted in front of the *LUC* gene to construct *ProMdCHS-LUC* and *ProMdUFGT-LUC* reporters. The full length *MdBBX47* was cloned and recombined downstream of the CaM35S promoter to construct *35 S::MdBBX47* effector (Fig. 7A, D). LUC activity assays revealed that co-transformation with *MdBBX47* significantly enhanced the promoter activities of *MdCHS* and *MdUFGT* compared to the control and empty vector (Fig. 7B, C, E, F). Therefore, MdBBX47 is a positive transcriptional regulator of *MdCHS* and *MdUFGT*, which can enhance their gene expression. Furthermore, we conducted GUS assays using the *ProMdCHS-GUS* and *ProMdUFGT-GUS* reporter vectors (Fig. 7G) for histochemical staining analysis. Transformation of *MdBBX47* significantly enhanced tobacco leaf GUS staining promoted by promoters of *MdCHS* or *MdUFGT* (Fig. 7H), with higher expression of *GUS* gene compared with the EV (Fig. 7I). Collectively, our findings indicate that MdBBX47 positively regulates anthocyanin biosynthesis by specifically binding to the G-box motifs in the promoters of *MdCHS* and *MdUFGT*.

### MdBBX47 activates the expression of MdMYB110a by binding G-Box-motif in its promoter

**Fig. 8:**
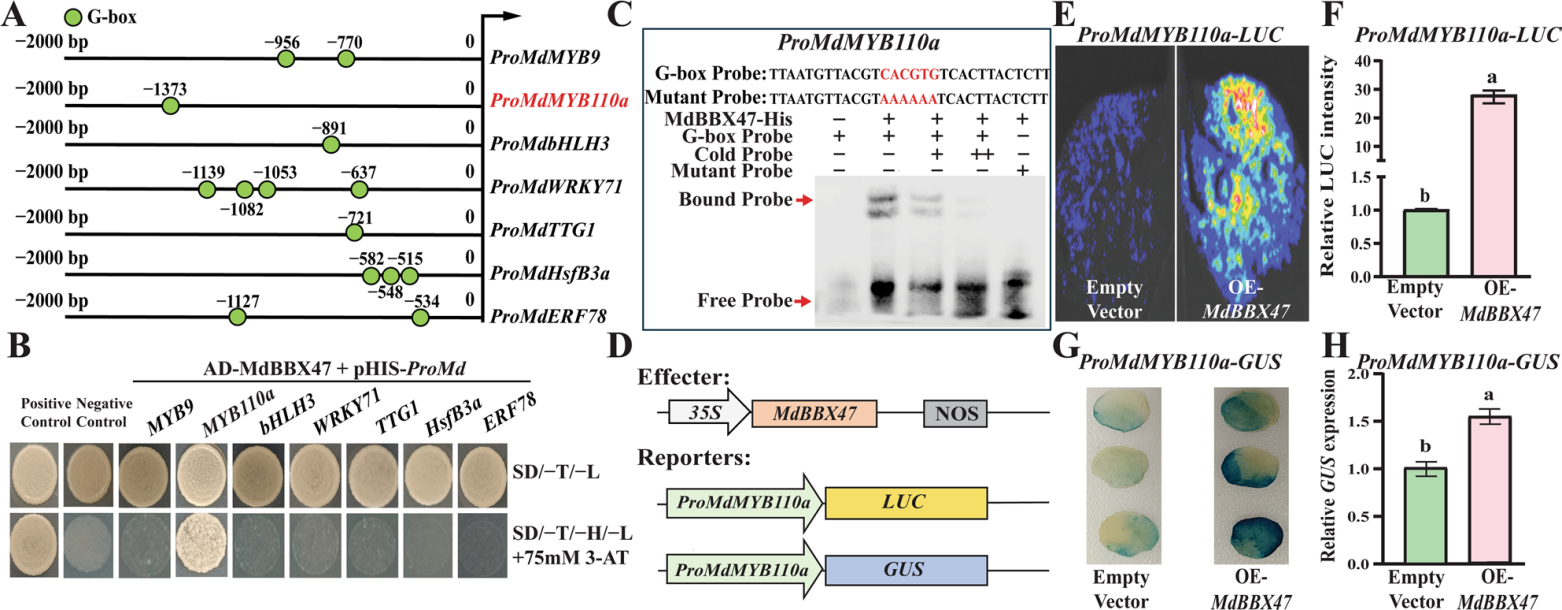
MdBBX47 transcriptionally regulates *MdMYB110a* expression activity. **A** Distribution of the G-box elements on promoters of seven transcription factor genes, where *proMdMYB110a* is marked in red color because it was studied in the subsequent experiments. **B** Y1H assays showing MdBBX47 binding to the promoters of *MdMYB9*, *MdMYB110a*,* MdbHLH3*,* MdWRKY71*,* MdTTG1*,* MdHsfB3a*, and *MdERF78*. Yeast harboring pGADT7-Rec2 and p53-pHIS2 plasmids served as the positive control, while pGADT7 and p53-pHIS2 as the negative control, SD − T/−L medium is the synthetic dropout medium lacking tryptophan and leucine, used for yeast transformation screening, SD − T/−H/−L represents synthetic dropout medium lacking leucine, tryptophan, and histidine, supplemented with 75 mM 3-AT. The Y1H assay confirmed that MdBBX47 binds only to the promoter of *MdMYB110a*. **C** EMSA results showing that MdBBX47-His binds to the G-box elements in the *MdMYB110a* promoter, using a biotin-labeled G-box-containing probe; –, absence of the relevant probes or proteins; +, presence of the relevant probes or proteins. **D** Schematic diagrams of the effector and reporter vectors used in the *LUC* and *GUS* reporter system. **E** Luminescence intensity in tobacco leaves indicates that MdBBX47 activates the *MdMYB110a* promoter. **F** Quantification of LUC fluorescence intensity. **G** GUS staining of tobacco leaves. **H** Relative *GUS* expression. Data are presented as means ± SE of three biological replicates, with different lowercase letters in each panel indicating significant differences (*P* < 0.05, Student’s *t-*test)

In addition to directly binding to the promoters of anthocyanins biosynthetic structural genes such as *MdCHS* and *MdUFGT*, MdBBX47 may regulate the transcription factor gene expression, indirectly mediating ALA-induced anthocyanin accumulation. To explore the possibility, we first examined the 2,000 bp promoter regions of the ALA-responsive TFs. It was found that seven anthocyanin-related TF genes, including *MdMYB9*, *MdMYB110a*, *MdbHLH3*, *MdWRKY71*, *MdTTG1*, *MdHsfB3a*, and *MdERF78*, harbor at least one G-box element (core sequence CACGTG) within the promoter regions (Fig. 8A), which represent potential binding sites for MdBBX TFs. Then, we employed a range of assays, including Y1H, EMSA, LUC and GUS assays to validate the possible binding. Y1H assays showed that only AD-MdBBX47 yeast cells can grow when *ProMdMYB110a*-*pHIS2* was co-transformed; conversely, those transformed with various promoter constructs, including *ProMdMYB9-pHIS2*, *ProMdbHLH3-pHIS2*, *ProMdWRKY71-pHIS2 ProMdTTG1-pHIS2*, *ProMdHsfB3a-pHIS2*, and *ProMdERF78-pHIS2* were unable to grow on selective medium SD/−Trp/−His/−Leu media supplemented with the corresponding concentration of 3-AT (Fig. 8B). These suggest that the TF can bind to the promoter of *MdMYB110a*, but not with the others such as *MdMYB9*, *MdbHLH3*, *MdWRKY71*, *MdTTG1*, *MdHsfB3a*, and *MdERF78*. EMSA further corroborated these findings, demonstrating that MdBBX47 directly and specifically interacted with the G-box motif in the *MdMYB110a* promoter. The binding affinity progressively decreased with increasing concentrations of cold probes, and no binding was detected with mutated probes (Fig. 8C). These results indicate that the G-box elements are crucial for MdBBX47 binding to the *MdMYB110a* promoter. Furthermore, we validated the transcriptional activation function of MdBBX47 on downstream *MdMYB110a* using LUC and GUS assays. Initially, we constructed the effector vector *35 S::MdBBX47* and reporter vector *ProMdMYB110a-*LUC *and ProMdMYB110a-*GUS (Fig. 8D). LUC assays demonstrated that MdBBX47 significantly elevated *MdMYB110a* promoter activity, as indicated by a marked increase in LUC signal compared to the empty vector control (Fig. 8E, F). Similarly, GUS reporter assays confirmed that MdBBX47 activated the *MdMYB110a* promoter, resulting in visible GUS staining and a higher *GUS* expression level in *MdBBX47*-overexpressing samples (Fig. 8G, H). Collectively, these results demonstrate that MdBBX47 directly binds to the G-box element in the *MdMYB110a* promoter and activates its transcription, highlighting the crucial role of MdBBX47 in regulating anthocyanin biosynthesis.

## Discussion

Given the importance of anthocyanins in producing high-quality fruit, substantial attention has been directed toward strategies to enhance their accumulation and elucidate the underlying regulatory mechanisms [52]. 5-Aminolevulinic acid (ALA) is a non-protein δ-amino acid and a pivotal precursor in tetrapyrrole biosynthesis [54, 55]. It is also recognized as a natural plant growth regulator with a broad range of biological effects [12], influencing the growth and development of various plant organs, including roots, stems, leaves, flowers, and fruits, under both optimal and stressful conditions [13]. Recently, studies on ALA have focused on the transcriptional regulatory mechanisms.

ALA has been shown to promote anthocyanin accumulation in various fruit species, such as apple [14, 20, 22, 28], litchi [15], peach [16], mango [56], grape [18, 57], and pear [17]. Therefore, it can be potentially applied for high-quality fruit production. Furthermore, the mechanisms for ALA to promote anthocyanin accumulation in apples has also been extensively explored [19]. It is well established that ALA enhances the expression of structural genes involved in anthocyanin biosynthesis, such as *MdCHS*, *MdF3H*, *MdDFR*, *MdANS*, and *MdUFGT*, the transport genes, such as *MdGSTF12* [24] and *MdMATE8* [4], as well as the transcription factor genes, such as *MdbHLH33*, *MdTTG1* [43], *MdMYB9*, *MdMYB11* [58], *MdMADS1* [22], *MdNAC33* [26], *MdERF78* [23], *MdMYB110a*, *MdWD40-280*, *MdHsfB3a* [24], and *MdWRKY71* [27]. Obviously, involvement of so many TF genes indicate the regulatory mechanisms behind ALA-induced apple anthocyanin accumulation are complex. Up to now, BBX transcription factors have not been reported involved in the process of ALA-induced anthocyanin accumulation.

In present study, we analyzed the phylogenetic relationship of apple BBX family members and observed that 64 members can be divided into five groups (Fig. 2A), which is consistent with the previous reports [30, 59]. Among them, we noticed that *MdBBX47*, clustered in Group II, is the most responsive factor to exogenous ALA in the Group (Fig. 2B). Phylogenetic tree analysis among different BBX members across species showed that MdBBX47 is very close to apple MdBBX20 as well as MaBBX20 of grape hyacinth (Fig. 3A). Alignment of the amino acid sequences shows that the three BBXs possess two B-Box domains at the N-terminal and a CCT-domain at the C-terminal, with similarity of 77.99% (Fig. 3B). However, the gene structure, conserved motifs, and domains of 10 MdBBX members of Group II are rather different from each other (Fig. S2). Analysis of the *cis*-elements in the promoters the ten BBX members has also not found obvious law (Fig. S3). Therefore, the conserved motifs of BBX members rather than the whole sequences are important for the protein function. Since apple *MdBBX20* [38] and grape hyacinth *MaBBX20* [51] are reported involved in anthocyanin accumulation, we decided to choose *MdBBX47* as the target gene to explore its possible function in ALA-induced anthocyanin accumulation.

In the actual experiments, we found that whether in apple fruits, calli or detached leaves, ALA treatment stimulated anthocyanin accumulation (Fig. 1) was always companied with upregulation of *MdBBX47* expression (Fig. 2B, C). With GUS staining method, we proved that ALA positively regulates *MdBBX47* gene expression by activating the transcriptional activity of the promoter (Fig. 2D). We further verified that MdBBX47 protein is localized in the nucleus (Fig. 3C), which is a basic property of transcription factor. Then, we employed transgenic apple to validate *MdBBX47* gene function in ALA-induced anthocyanin accumulation. Whether in apple fruits or detached leaves, OE-*MdBBX47* promoted anthocyanin accumulation, whereas RNAi-*MdBBX47* depressed the accumulation (Fig. 4A and D). The responses were approved in apple calli (Fig. 5A). Moreover, ALA further promoted the effect of OE-*MdBBX47* but without rescue effects in the RNAi lines. These findings indicate that MdBBX47 is a crucial transcription factor for ALA-induced apple anthocyanin biosynthesis. Up to now, it is the first report to show that MdBBX47 is involved in ALA-induced apple anthocyanin accumulation.

Several ways have been suggested for ALA-induced TFs to regulate anthocyanin biosynthesis. Firstly, the TFs directly bind to the *cis*-elements within the promoters of the structural genes related to anthocyanin biosynthesis or transport to promote gene transcription. Secondly, the TFs bind to the *cis*-elements of other TF gene promoters, then the latter transcriptionally regulate anthocyanin biosynthesis gene expression, indirectly promoting anthocyanin accumulation. Thirdly, ALA promoted TF gene expression, then the TF interacting with other TF at protein levels to form regulatory complexes to regulate downstream gene transcription and anthocyanin accumulation. For example, ALA-induced MdERF78 can directly bind to the promoters of *MdF3H* and *MdANS* to promote anthocyanin biosynthesis. It can also interact with MdMYB1 to bind to the promoters of *MdDFR*, *MdUFGT* and *MdGSTF12* to promote anthocyanin accumulation [24]. MdNAC33, another ALA-induced TF, can directly bind to the promoters of *MdDFR* and *MdANS* to promote anthocyanin biosynthesis. It also upregulates *MdbHLH3* expression, then MdbHLH3 transcriptionally regulates *MdDFR* and *MdUFGT* expression to promote anthocyanin biosynthesis. Additionally, MdNAC33 can interact with MdMYB1, strengthening the binding affinity of the latter to the promoters of *MdUFGT* and *MdGSTF12*, consequently, promoting anthocyanin accumulation [26]. A pair of TFs, MdWRKY71 and MdMADS1, are induced by ALA, jointly transcriptionally regulating ALA-promoted anthocyanin accumulation. Initially, MdWRKY71 transcriptionally regulates *MdDFR* and *MdANS* expression, directly promoting anthocyanin biosynthesis. Furthermore, MdWRKY71 transcriptionally regulates *MdMADS1* expression, then MdMADS1 binds to the promoters of *MdCHS* and *MdUFGT*, indirectly promoting anthocyanin biosynthesis. Finally, MdWRKY71 and MdMADS1 interact with each other to form a heterodimer, acting in two different ways. Firstly, MdMADS1 assists MdWRKY71 to enhance the latter’s transcriptional regulatory activity on *MdDFR* and *MdANS*; secondly, MdWRKY71 assists MdMADS1 to enhance the latter’s transcriptional regulatory activity on *MdCHS* and *MdUFGT*, jointly promoting anthocyanin accumulation [27]. The fine regulatory mechanism has never been reported before. Additionally, MdMYB110a has been verified as an ALA-induced transcription factor that targets and upregulates the expression of MdDFR, MdANS, and MdGSTF12, thereby promoting anthocyanin accumulation [24]. Up to now, however, the mechanism by which ALA-induced MdBBX47 regulates anthocyanin accumulation has rarely been reported.

In present study, we performed promoter analysis, Y1H, EMSA (Fig. 6), LUC fluorescence, and GUS staining (Fig. 7) to verify that MdBBX47 can bind to the promoters of *MdCHS* and *MdUFGT*, therefore, directly promoting anthocyanin biosynthesis. It is consistent with PavBBX6/9 in sweet cherry (*Prunus avium*), which activate *PavUFGT* by directly binding to G-box element of the promoter [49]. However, in pear (*Pyrus pyrifolia*), PpBBX16 does not directly activate structural gene expression but interact with PpHY5 to activate *PpMYB10* expression, indirectly promoting anthocyanin biosynthesis [60]. Similarly, PpBBX18 and PpBBX21 competitively interact with PpHY5, indirectly affecting anthocyanin accumulation in pear [61]. In grapevine (*Vitis vinifera*), VvBBX44 represses *VvUFGT*, *VvMYBA1*, and *VvHY5* expression, directly and indirectly inhibits anthocyanin accumulation [62]. In apple, many MdBBXs are reported involved in anthocyanin accumulation. For example, MdBBX1/15/35/51/54 as activators of *MdMYB10* promoter, indirectly promote *MdDFR* expression and anthocyanin accumulation [59]. MdBBX20, as a positive factor binds to the promoters of *MdDFR*, *MdANS*, and *MdMYB1*, directly and indirectly promote anthocyanin synthesis [38]. In the present study, we found that ALA-induced MdBBX47 can affect many TF gene expression, such as *MdMYB1*/*9*/*110a*, *MdTTG1*, *MdNAC33*, *MdEFR78*, *MdHsfB3a* and *MdWRKY71* (Fig. 5D), however, further study showed that only *MdMYB110a* expression was transcriptionally regulated by MdBBX47 (Fig. 8). These suggest that MdBBX47 can promote anthocyanin biosynthesis by directly binding to promoters of anthocyanin biosynthetic genes, as well as upregulate *MdMYB110a* expression to indirectly promote anthocyanin accumulation. In addition, MdHY5 is also an important TF mediating light signaling and regulating growth and development [39, 63, 64]. HY5 transcription factor is recognized as a central positive regulator of light signaling and it has been previously reported that MdHY5 is an ALA-responsive BBX member [65]. By Y2H, we found that MdBBX47 can physically interact with MdHY5. LCI assay confirms the interaction between MdBBX47 and MdHY5 (Fig. S4). Therefore, MdBBX47 may interact with MdHY5 to regulate anthocyanin accumulation through MBW route, which should be studied further.

## Conclusion

**Fig. 9:**
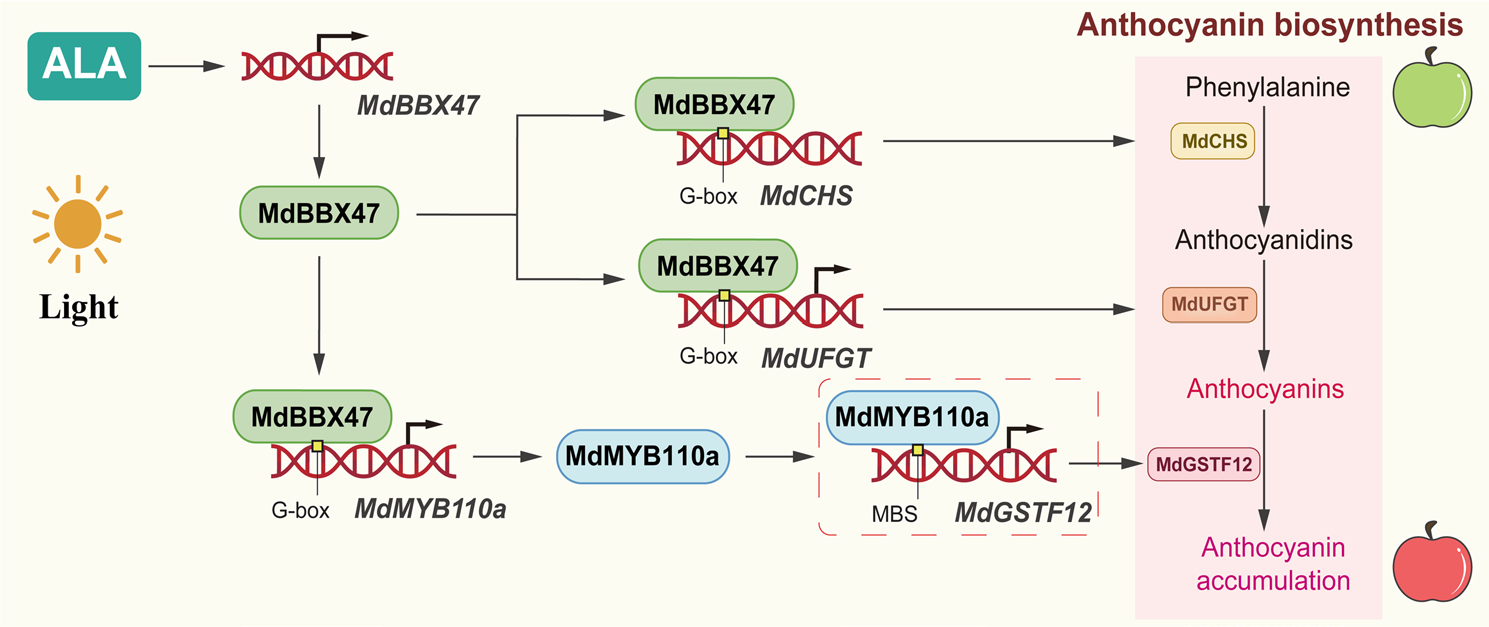
In the proposed regulatory model, the transcription factor MdBBX47 mediates ALA-induced anthocyanin accumulation by binding to G-box elements in the promoters of genes such as *MdCHS* and *MdUFGT*, to directly promote anthocyanin biosynthesis. Additionally, MdBBX47 can enhance the expression of *MdMYB110a*, which further transcriptionally upregulates the transport gene *MdGSTF12* expression, indirectly promoting anthocyanin accumulation

Based on the findings in the study, we propose a regulatory model illustrating the function of MdBBX47 in ALA-induced anthocyanin accumulation (Fig. 9). This model advances our understanding of the molecular mechanism underlying ALA-induced pigmentation and highlights the significant role of the MdBBX47 regulatory module.

## Supplementary Information

Supplementary Material 1.

Supplementary Material 2.

Supplementary Material 3.

Supplementary Material 4.

## Data Availability

The datasets used or analyzed during the current study are available from the corresponding author on reasonable request.

## References

[1] He J, Giusti MM. Anthocyanins: natural colorants with health-promoting properties. Annu Rev Food Sci Technol. 2010;1:163–87. 22129334 10.1146/annurev.food.080708.100754

[2] Li H, Deng K, Zhao Y, Xu D. A comprehensive review of BBX protein-mediated regulation of anthocyanin biosynthesis in horticultural plants. Horticulturae. 2025;11(8):894.

[3] Winkel-Shirley B. Flavonoid biosynthesis. A colorful model for genetics, biochemistry, cell biology, and biotechnology. Plant Physiol. 2001;126(2):485–93. 11402179 10.1104/pp.126.2.485 PMC1540115

[4] Zheng J, Liu L, Tao H, An Y, Wang L. Transcriptomic profiling of apple calli with a focus on the key genes for ALA-induced anthocyanin accumulation. Front Plant Sci. 2021;12:640606. 33841467 10.3389/fpls.2021.640606 PMC8033201

[5] Kim SH, Lee JR, Hong ST, Yoo YK, An G, Kim SR. Molecular cloning and analysis of anthocyanin biosynthesis genes preferentially expressed in apple skin. Plant Sci. 2003;165(2):403–13.

[6] Zhang H, Jordheim M, Lewis DH, Arathoon S, Andersen ØM, Davies KM. Anthocyanins and their differential accumulation in the floral and vegetative tissues of a shrub species (*Rhabdothamnus solandri* A. Cunn). Sci Hort. 2014;165:29–35.

[7] Allan AC, Espley RV. MYBs drive novel consumer traits in fruits and vegetables. Trends Plant Sci. 2018;23(8):693–705. 30033210 10.1016/j.tplants.2018.06.001

[8] Espley RV, Hellens RP, Putterill J, Stevenson DE, Kutty-Amma S, Allan AC. Red colouration in apple fruit is due to the activity of the MYB transcription factor, MdMYB10. Plant J. 2007;49(3):414–27. 17181777 10.1111/j.1365-313X.2006.02964.x PMC1865000

[9] Bai S, Saito T, Honda C, Hatsuyama Y, Ito A, Moriguchi T. An apple B-box protein, MdCOL11, is involved in UV-B- and temperature-induced anthocyanin biosynthesis. Planta. 2014;240(5):1051–62. 25074586 10.1007/s00425-014-2129-8

[10] Takos AM, Jaffé FW, Jacob SR, Bogs J, Robinson SP, Walker AR. Light-induced expression of a MYB gene regulates anthocyanin biosynthesis in red apples. Plant Physiol. 2006;142(3):1216–32. 17012405 10.1104/pp.106.088104 PMC1630764

[11] Xie XB, Li S, Zhang RF, Zhao J, Chen YC, Zhao Q, Yao YX, You CX, Zhang XS, Hao YJ. The bHLH transcription factor MdbHLH3 promotes anthocyanin accumulation and fruit colouration in response to low temperature in apples. Plant Cell Environ. 2012;35(11):1884–97. 22519753 10.1111/j.1365-3040.2012.02523.x

[12] Wang L, Zhang J, Zhong Y, Zhang L, Yang H, Liu L, Zhou J, Iqbal M, Gan X. Regulation of 5-aminolevunilic acid and its application in agroforestry. Forests. 2023a;14(9):1857.

[13] Wang L, Zhang J, Zhao Q, Zhang L. 5-Aminolevulinic acid: from pyrrole biosynthetic precursor to multifunctional plant growth regulator. J Plant Physiol. 2025;310:154524. 40398357 10.1016/j.jplph.2025.154524

[14] Wang L, Wang Z, Li Z, Liu H, Liu W, Chen Z, Yan P, Sun D. Effect of 5-aminolevulinic acid on enhancing apple fruit coloration. J Fruit Sci. 2004;21(6):512–5.

[15] Feng S, Li M, Wu F, Li W, Li S. 5-Aminolevulinic acid affects fruit coloration, growth, and nutrition quality of *Litchi chinensis* Sonn. cv. Feizixiao in Hainan, tropical China. Sci Hort. 2015;193:188–94.

[16] Ye J, Yang X, Chen Q, Xu F, Wang G. Promotive effects of 5-aminolevulinic acid on fruit quality and coloration of *Prunus persica* (L.) Batsch. Sci Hort. 2017;217:266–75.

[17] Cao X, Sun H, Wang X, Li W, Wang X. ABA signaling mediates 5-aminolevulinic acid-induced anthocyanin biosynthesis in red pear fruits. Sci Hort. 2022;304:111290.

[18] Zhang Z, Liu L, Chang X, He W, Liu J, Zhao B, Sun J. Effects of 5-aminolevulinic acid on Anthocyanin synthesis in *Vitis Vinifera* ‘Crimson Seedless’ grapes at the transcriptomics level. J Hortic Sci Biotech. 2021;96(6):797–807.

[19] Xie L, Wang Z, Cheng X, Gao J, Zhang Z, Wang L. 5-Aminolevulinic acid promotes anthocyanin accumulation in Fuji apples. Plant Growth Regul. 2013;69(3):295–303.

[20] Zheng J, An Y, Wang L. 24-Epibrassinolide enhances 5-ALA-induced anthocyanin and flavonol accumulation in calli of ‘Fuji’apple flesh. Plant Cell Tiss Organ Cult. 2018;134:319–30.

[21] Fang X, An Y, Zheng J, Shangguan L, Wang L. Genome–wide identification and comparative analysis of GST gene family in apple (*Malus domestica*) and their expressions under ALA treatment. 3 Biotech. 2020;10:307. 32582504 10.1007/s13205-020-02299-x PMC7295890

[22] Feng X, An Y, Zheng J, Sun M, Wang L. Proteomics and SSH analyses of ALA-promoted fruit coloration and evidence for the involvement of a MADS-box gene, *MdMADS1*. Front Plant Sci. 2016;7:1615. 27872628 10.3389/fpls.2016.01615 PMC5098116

[23] Fang X, Zhang L, Wang L. The transcription factor MdERF78 is involved in ALA-induced anthocyanin accumulation in apples. Front Plant Sci. 2022;13:915197. 35720608 10.3389/fpls.2022.915197 PMC9201628

[24] Fang X, Zhang l, Shangguan L, Wang L. MdMYB110a, directly and indirectly, activates the structural genes for the ALA-induced accumulation of anthocyanin in apple. Plant Sci. 2023;326:111511. 36377142 10.1016/j.plantsci.2022.111511

[25] Chagné D, Lin-Wang K, Espley RV, Volz RK, How NM, Rouse S, Brendolise C, Carlisle CM, Kumar S, De Silva N, Micheletti D, McGhie T, Crowhurst RN, Storey RD, Velasco R, Hellens RP, Gardiner SE, Allan AC. An ancient duplication of apple MYB transcription factors is responsible for novel red fruit-flesh phenotypes. Plant Physiol. 2013;161(1):225–39. 23096157 10.1104/pp.112.206771 PMC3532254

[26] Zhang L, Zhang J, Wei B, Li Y, Fang X, Zhong Y, Wang L. Transcription factor MdNAC33 is involved in ALA-induced anthocyanin accumulation in apples. Plant Sci. 2024;339:111949. 38065304 10.1016/j.plantsci.2023.111949

[27] Zhang L, Tao H, Zhang J, An Y, Wang L. 5-Aminolevulinic acid activates the MdWRKY71-MdMADS1 module to enhance anthocyanin biosynthesis in apple. Mol Hortic. 2025;5(1):10. 39894860 10.1186/s43897-024-00127-x PMC11789342

[28] Yin Y, Zhang L, Zhang J, Zhong Y, Wang L. *MdFC2*, a ferrochelatase gene, is a positive regulator of ALA-induced anthocyanin accumulation in apples. J Plant Physiol. 2025;304:154381. 39612779 10.1016/j.jplph.2024.154381

[29] Gangappa SN, Botto JF. The BBX family of plant transcription factors. Trends Plant Sci. 2014;19(7):460–70. 24582145 10.1016/j.tplants.2014.01.010

[30] Liu X, Li R, Dai YQ, Chen XS, Wang XY. Genome-wide identification and expression analysis of the B-box gene family in the apple (*Malus domestica* Borkh.) genome. Mol Genet Genomics. 2018;293:303–15. 29063961 10.1007/s00438-017-1386-1

[31] Cao Y, Han Y, Meng D, Li D, Jiao C, Jin Q, Lin Y, Cai Y. B-BOX genes: genome-wide identification, evolution and their contribution to pollen growth in pear (*Pyrus bretschneideri Rehd*). BMC Plant Biol. 2017;17(1):156. 28927374 10.1186/s12870-017-1105-4 PMC5606111

[32] Zou Z, Wang R, Wang R, Yang S, Yang Y. Genome-wide identification, phylogenetic analysis, and expression profiling of the BBX family genes in pear. J Hortic Sci Biotech. 2018;93(1):37–50.

[33] Khanna R, Kronmiller B, Maszle DR, Coupland G, Holm M, Mizuno T, Wu SH. The Arabidopsis B-box zinc finger family. Plant Cell. 2009;21(11):3416–20. 19920209 10.1105/tpc.109.069088 PMC2798317

[34] Huang J, Zhao X, Weng X, Wang L, Xie W. The rice B-box zinc finger gene family: genomic identification, characterization, expression profiling and diurnal analysis. PLoS ONE. 2012;7(10):Pe48242. 10.1371/journal.pone.0048242 PMC3485221 23118960

[35] Talar U, Kiełbowicz-Matuk A, Czarnecka J, Rorat T. Genome-wide survey of B-box proteins in potato (*Solanum tuberosum*)-identification, characterization and expression patterns during diurnal cycle, etiolation and de-etiolation. PLoS ONE. 2017;12(5):e0177471. 28552939 10.1371/journal.pone.0177471 PMC5446133

[36] Chu Z, Wang X, Li Y, Yu H, Li J, Lu Y, Li H, Ouyang B. Genomic organization, phylogenetic and expression analysis of the B-BOX gene family in tomato. Front Plant Sci. 2016;7:1552. 27807440 10.3389/fpls.2016.01552 PMC5069294

[37] Wei H, Wang P, Chen J, Li C, Wang Y, Yuan Y, Fang J, Leng X. Genome-wide identification and analysis of B-BOX gene family in grapevine reveal its potential functions in berry development. BMC Plant Biol. 2020;20(1):72. 32054455 10.1186/s12870-020-2239-3 PMC7020368

[38] Fang H, Dong Y, Yue X, Hu J, Jiang S, Xu H, Wang Y, Su M, Zhang J, Zhang Z, Wang N, Chen X. The B-box zinc finger protein MdBBX20 integrates anthocyanin accumulation in response to ultraviolet radiation and low temperature. Plant Cell Environ. 2019b;42(7):2090–104. 30919454 10.1111/pce.13552

[39] An JP, Wang XF, Espley RV, Lin-Wang K, Bi SQ, You CX, Hao YJ. An apple B-Box protein MdBBX37 modulates anthocyanin biosynthesis and hypocotyl elongation synergistically with MdMYBs and MdHY5. Plant Cell Physiol. 2020b;61(1):130–43. 31550006 10.1093/pcp/pcz185

[40] An JP, Qu FJ, Yao JF, Wang XN, You CX, Wang XF, et al. The bZIP transcription factor MdHY5 regulates anthocyanin accumulation and nitrate assimilation in apple. Hortic Res. 2017;4:17023. 28611922 10.1038/hortres.2017.23 PMC5461414

[41] Livak KJ, Schmittgen TD. Analysis of relative gene expression data using real-time quantitative PCR and the 2^–∆∆CT^ method. Methods. 2001;25(4):402–8. 11846609 10.1006/meth.2001.1262

[42] Chen Z, An YY, Wang LJ. ALA reverses ABA-induced stomatal closure by modulating PP2AC and SnRK2.6 activity in apple leaves. Hortic Res. 2023;10(6):uhad067. 37287446 10.1093/hr/uhad067 PMC10243991

[43] Chen Z, Wang LJ. ALA upregulates *MdPTPA* expression to increase the PP2A activity and promote stomatal opening in apple leaves. Plant Sci. 2022;325:111490. 36216297 10.1016/j.plantsci.2022.111490

[44] Zheng J, An Y, Feng X, Wang L. Rhizospheric application with 5-aminolevulinic acid improves coloration and quality in ‘Fuji’ apples. Sci Hort. 2017;224:74–83.

[45] Chang CSJ, Maloof JN, Wu SH. COP1-mediated degradation of BBX22/LZF1 optimizes seedling development in Arabidopsis. Plant Physiol. 2011;156:228–39. 21427283 10.1104/pp.111.175042 PMC3091042

[46] Bursch K, Toledo-Ortiz G, Pireyre M, Lohr M, Braatz C, Johansson H. Identification of BBX proteins as rate-limiting cofactors of HY5. Nat Plants. 2020;6(8):921–8. 32661279 10.1038/s41477-020-0725-0

[47] An JP, Wang XF, Zhang XW, Bi SQ, You CX, Hao YJ. MdBBX22 regulates UV-B-induced anthocyanin biosynthesis through regulating the function of MdHY5 and is targeted by MdBT2 for 26S proteasome-mediated degradation. Plant Biotechnol J. 2019;17(12):2231–3. 31222855 10.1111/pbi.13196 PMC6835122

[48] Liu L, Zheng J. Identification and characterization of the BBX gene family in pomegranate (*Punica granatum* L.) and its potential role in anthocyanin accumulation during fruit ripening. Horticulturae. 2025;11(5):507.

[49] Wang Y, Xiao Y, Sun Y, Zhang X, Du B, Turupu M, Yao Q, Gai S, Tong S, Huang J, Li T. Two B-box proteins, PavBBX6/9, positively regulate light-induced anthocyanin accumulation in sweet cherry. Plant Physiol. 2023b;192(3):2030–48. 36930566 10.1093/plphys/kiad137 PMC10315283

[50] Luo D, Xiong C, Lin A, Zhang C, Sun W, Zhang J, Yang C, Lu Y, Li H, Ye Z, He P, Wang T. SlBBX20 interacts with the COP9 signalosome subunit SlCSN5-2 to regulate anthocyanin biosynthesis by activating SlDFR expression in tomato. Hortic Res. 2021;8(1):163. 34193855 10.1038/s41438-021-00595-y PMC8245592

[51] Zhang H, Wang J, Tian S, Hao W, Du L. Two B-box proteins, MaBBX20 and MaBBX51, coordinate light-induced anthocyanin biosynthesis in grape hyacinth. Int J Mol Sci. 2022;23:5678. 10.3390/ijms23105678 PMC9146254 35628488

[52] Liu Y, Ye Y, Wang Y, Jiang L, Yue M, Tang L, Jin M, Zhang Y, Lin Y, Tang H. B-box transcription factor FaBBX22 promotes light-induced anthocyanin accumulation in strawberry (*Fragaria × ananassa*). Int J Mol Sci. 2022;23(14):7757. 35887106 10.3390/ijms23147757 PMC9316111

[53] Zhang L, Wang Y, Yue M, Jiang L, Zhang N, Luo Y, Chen Q, Zhang Y, Wang Y, Li M. FaMYB5 interacts with FaBBX24 to regulate anthocyanin and proanthocyanidin biosynthesis in strawberry (*Fragaria× ananassa*). Int J Mol Sci. 2023;24(15):12185. 37569565 10.3390/ijms241512185 PMC10418308

[54] Akram NA, Ashraf M. Regulation in plant stress tolerance by a potential plant growth regulator, 5-aminolevulinic acid. J Plant Growth Regul. 2013;32:663–79.

[55] Wu Y, Liao W, Dawuda MM, Hu L, Yu J. 5-Aminolevulinic acid (ALA) biosynthetic and metabolic pathways and its role in higher plants: a review. Plant Growth Regul. 2019;87(2):357–74.

[56] Meng FG, Duan YJ, Yang TJ, Lv SY, Liu HG. Effect of 5-aminolevulinic acid treatment on physicochemical characteristics and color development of mango fruit. South China Fruit Trees. 2019;48(3):57–6.

[57] Aziz RB, Wei J, Wu Q, Song S, Yang H, Chen X, Wang Y, Chao R, Baz NM, Chen H, Song Y, Fang J, Wang C. Characterization of main responsive genes reveals their regulatory network attended by multi-biological metabolic pathways in paclobutrazol (PAC)-modulated grape seed development (GSD) at the stone-hardening stage. Int J Mol Sci. 2025;26(3):1102. 39940872 10.3390/ijms26031102 PMC11817196

[58] An XH, Tian Y, Chen KQ, Liu XJ, Liu DD, Xie XB, Cheng CG, Cong PH, Hao YJ. MdMYB9 and MdMYB11 are involved in the regulation of the JA-induced biosynthesis of anthocyanin and proanthocyanidin in apples. Plant Cell Physiol. 2015;56(4):650–62. 25527830 10.1093/pcp/pcu205

[59] Plunkett BJ, Henry-Kirk R, Friend A, Diack R, Helbig S, Mouhu K, Tomes S, Dare AP, Espley RV, Putterill J, Allan AC. Apple B-box factors regulate light-responsive anthocyanin biosynthesis genes. Sci Rep. 2019;9(1):17762. 31780719 10.1038/s41598-019-54166-2 PMC6882830

[60] Bai S, Tao R, Tang Y, Yin L, Ma Y, Ni J, Yan X, Yang Q, Wu Z, Zeng Y, Teng Y. BBX16, a B-box protein, positively regulates light-induced anthocyanin accumulation by activating *MYB10* in red pear. Plant Biotechnol J. 2019a;17(10):1985–97. 30963689 10.1111/pbi.13114 PMC6737026

[61] Bai S, Tao R, Yin L, Ni J, Yang Q, Yan X, Yang F, Guo X, Li H, Teng Y. Two B-box proteins, PpBBX18 and PpBBX21, antagonistically regulate anthocyanin biosynthesis via competitive association with *Pyrus pyrifolia* ELONGATED HYPOCOTYL 5 in the peel of pear fruit. Plant J. 2019b;100(6):1208–23. 31444818 10.1111/tpj.14510

[62] Liu W, Mu H, Yuan L, Li Y, Li Y, Li S, Ren C, Duan W, Fan P, Dai Z, Zhou Y, Liang Z, Li S, Wang L. VvBBX44 and VvMYBA1 form a regulatory feedback loop to balance anthocyanin biosynthesis in grape. Hortic Res. 2023;10(10):uhad176. 37868620 10.1093/hr/uhad176 PMC10585713

[63] An JP, Liu YJ, Zhang XW, Bi SQ, Wang XF, You CX, Hao YJ. Dynamic regulation of anthocyanin biosynthesis at different light intensities by the BT2-TCP46-MYB1 module in apple. J Exp Bot. 2020a;71(10):3094–109. 31996900 10.1093/jxb/eraa056 PMC7475178

[64] Gangappa SN, Crocco CD, Johansson H, Datta S, Hettiarachchi C, Holm M, Botto JF. The Arabidopsis B-BOX protein BBX25 interacts with HY5, negatively regulating *BBX22* expression to suppress seedling photomorphogenesis. Plant Cell. 2013;25(4):1243–57. 23624715 10.1105/tpc.113.109751 PMC3663265

[65] Zhang H, Tao H, Yang H, Zhang L, Feng G, An Y, Wang L. MdSCL8 as a negative regulator participates in ALA-induced FLS1 to promote flavonol accumulation in apples. Int J Mol Sci. 2022a;23:2033. 10.3390/ijms23042033 PMC8875840 35216148

