# Supplementary material for: MdBBX47, a B-box transcription factor directly and indirectly regulates ALA-induced anthocyanin accumulation in apple
Source: BMC Plant Biol. 2026 Apr 29;26:1038. doi: 10.1186/s12870-026-08838-7 (PMC13270602; doi:10.1186/s12870-026-08838-7)
Supplement: Supplementary file 2 — Supplementary Material 2. [file 12870_2026_8838_MOESM2_ESM.docx]

**Supplementary data**

**Article title: MdBBX47, a B-box transcription factor directly and indirectly regulates ALA-induced anthocyanin accumulation in apple**


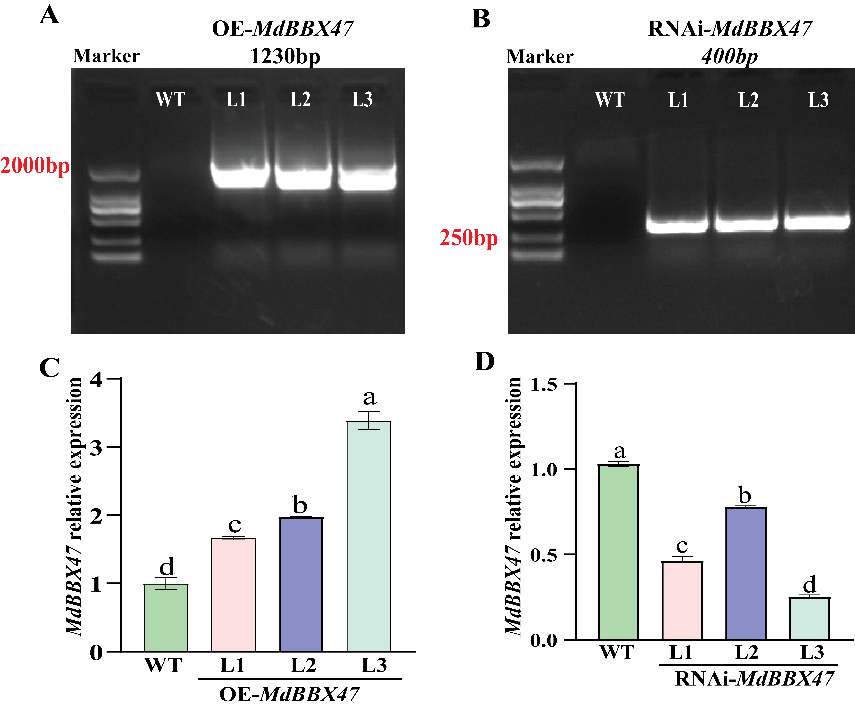


**Fig.S1. Identification of *MdBBX47* in the transformed ‘Orin’ apple calli by PCR and RT-qPCR. A** and **B:** Confirmation of transgenic lines by PCR amplification. **C** and **D:** The expressions of *MdBBX47* in different transgenic lines were determined by RT-qPCR. The data in the figure are means ± SE of three biological replicates. The different small letters in each panel represent significant differences (*P* < 0.05).


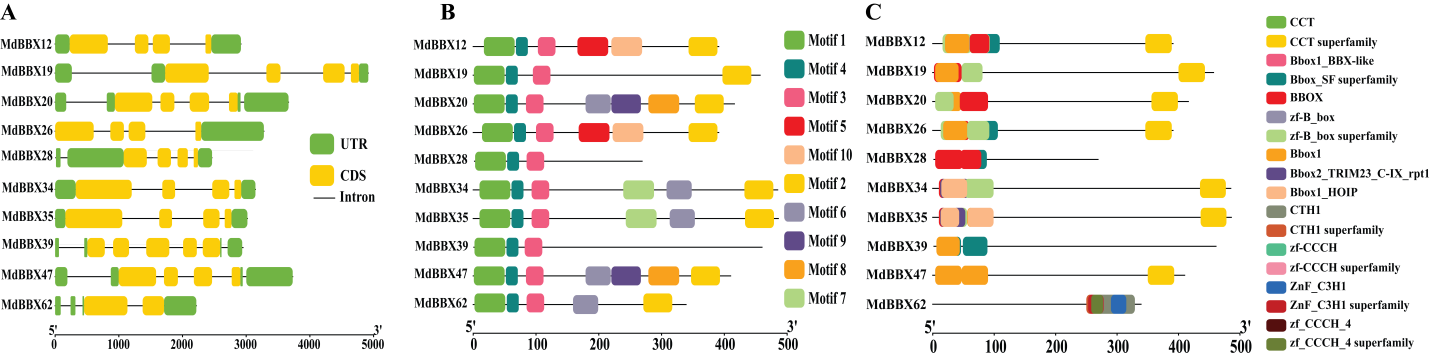


**Fig. S2 Analysis of the gene structure, conserved motifs, and domains of 10 members of Group II of *MdBBX* genes. (A)** Gene structure of *MdBBX* genes, where green boxes, yellow boxes and thin gray lines represent non-coding regions, exons and introns, respectively. **(B)** Conserved motifs of 10 MdBBX proteins, with a total of 10 conserved motifs, represented by boxes in different colors. **(C)** Conserved domains of 10 MdBBX proteins, represented by boxes in different colors.

**
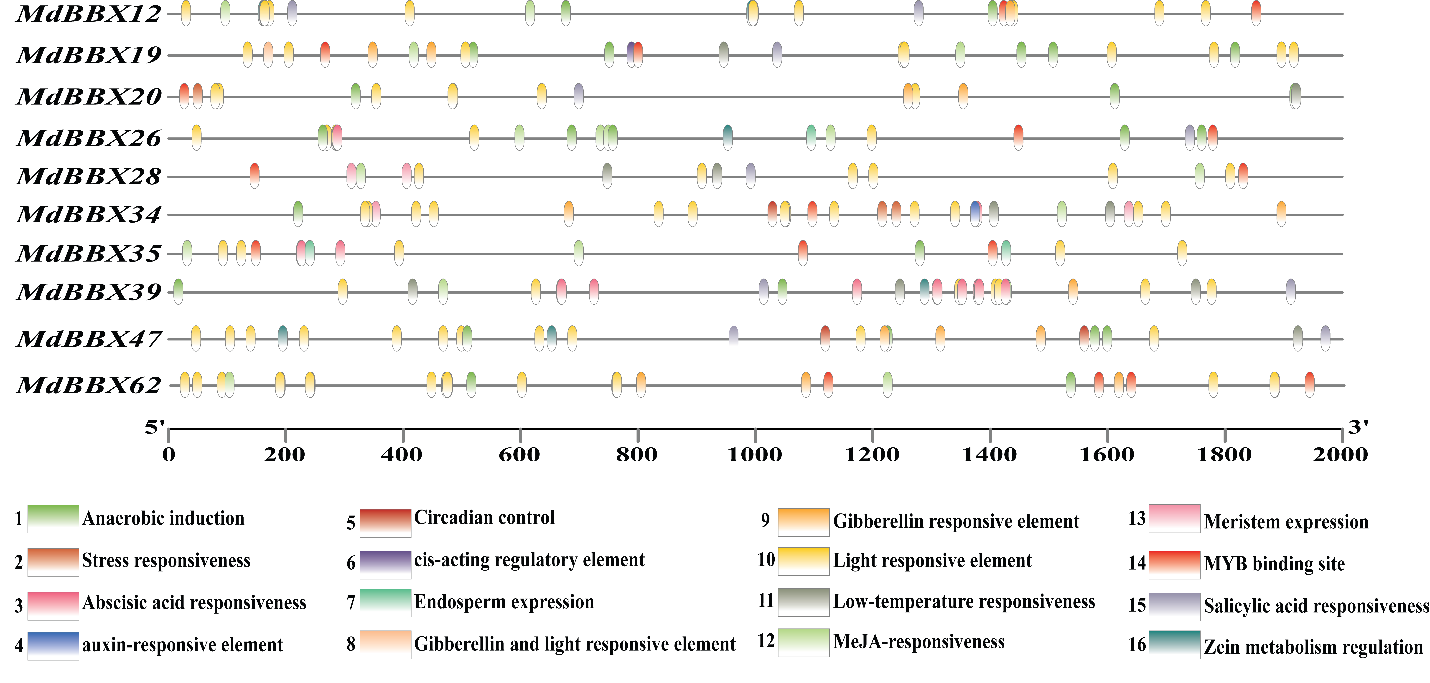
**

**Fig. S3 Analysis of cis-acting regulatory elements in the promoters of ten members of Group II in the apple *MdBBX* subfamily**.


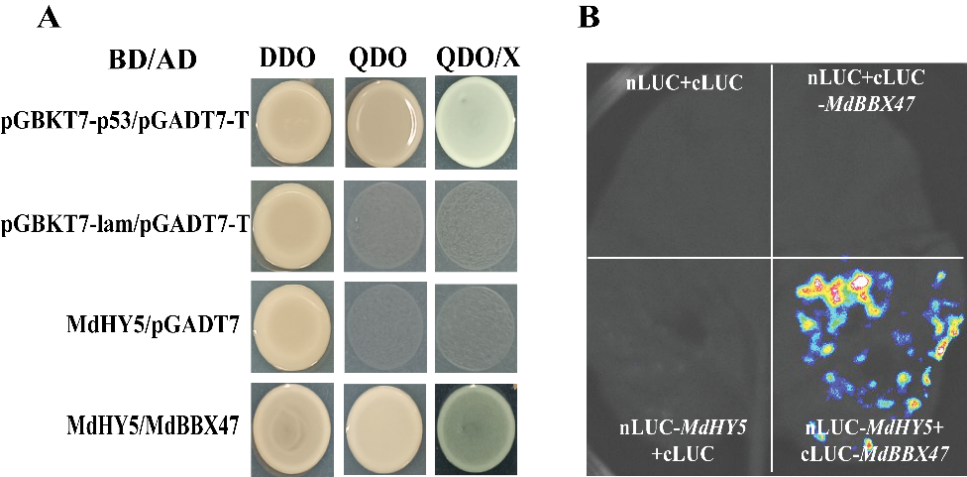


**Fig. S4 Molecular interaction between MdBBX47 and MdHY5.** (**A**) Y2H assay validates that MdHY5 interacts with MdBBX47. When the co-transformants of pGBKT7-MdHY5/pGADT7-MdBBX47 grew on QDO/X medium (SD/−Ade/−His/−Leu/−Trp/X-α-Gal), only MdHY5/MdBBX47 produced blue colonies, suggesting a strong interaction between two proteins. Positive control: pGBKT7-53/pGADT7-T. Negative controls: pGBKT7-Lam/pGADT7-T, pGBKT7-MdHY5/pGADT7. DDO: Double dropout medium, SD/−Leu/−Trp. QDO: Quarter dropout medium, SD/−Ade/-His/−Leu/−Trp. The concentration of 3-amino-1, 2, 4-triazole and X-α-galactoside. (**B**) Luciferase complementation imaging (LCI) assay confirms the interaction between MdBBX47 and MdHY5.
