# Supplementary material for: MdBBX47, a B-box transcription factor directly and indirectly regulates ALA-induced anthocyanin accumulation in apple
Source: BMC Plant Biol. 2026 Apr 29;26:1038. doi: 10.1186/s12870-026-08838-7 (PMC13270602; doi:10.1186/s12870-026-08838-7)
Supplement: Supplementary file 3 — Supplementary Material 3. [file 12870_2026_8838_MOESM3_ESM.docx]

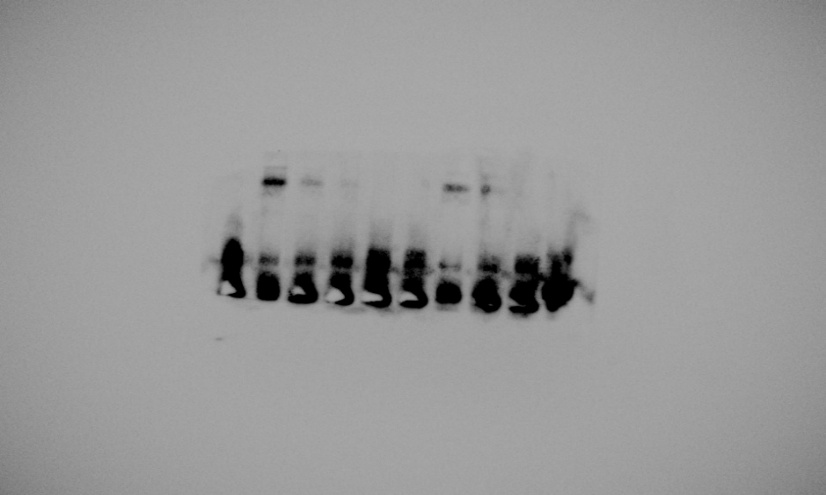


Fig. 6C: EMSA results showing that MdBBX47-His binds to the G-box elements in the *MdCHS* promoter, using a biotin-labeled G-box-containing probe; –, absence of the relevant probes or proteins; +, presence of the relevant probes or proteins.


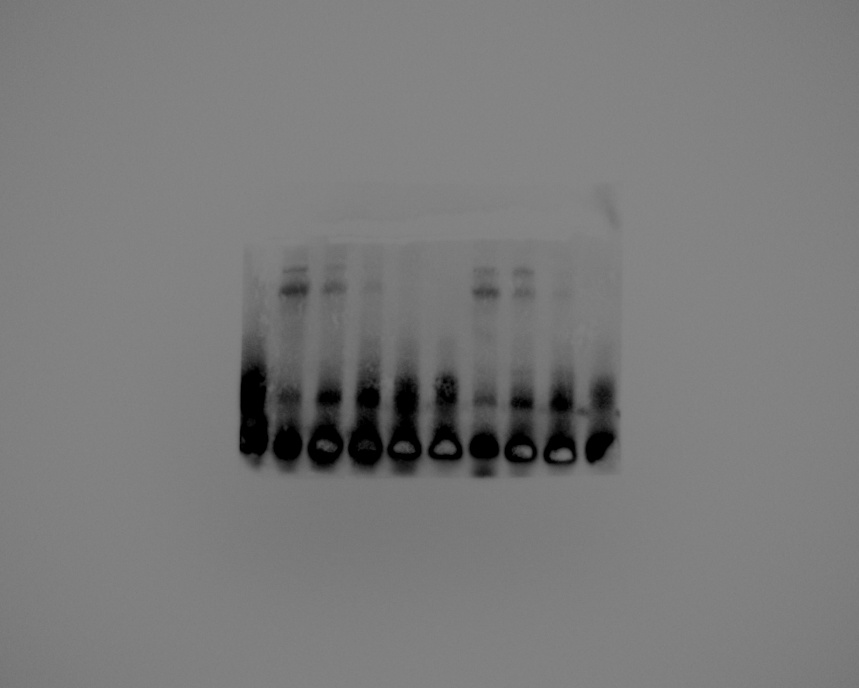


Fig. 6C: EMSA results showing that MdBBX47-His binds to the G-box elements in the *MdUFGT* promoter, using a biotin-labeled G-box-containing probe; –, absence of the relevant probes or proteins; +, presence of the relevant probes or proteins.


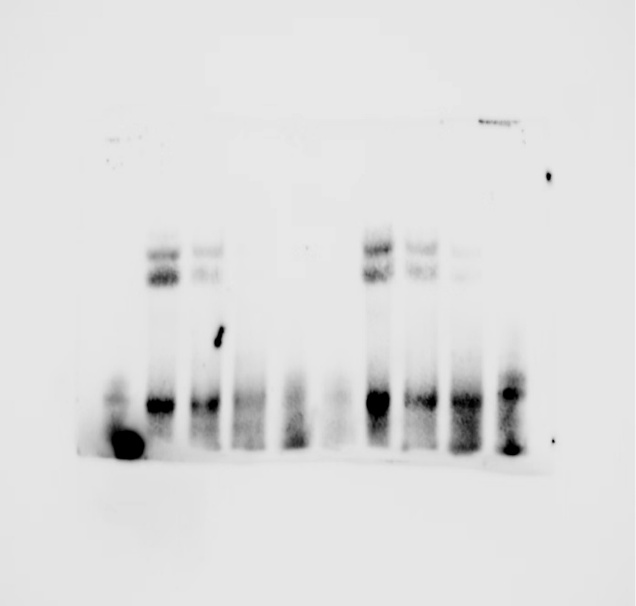


Fig. 8C: EMSA results showing that MdBBX47-His binds to the G-box elements in the *MdMYB110a* promoter, using a biotin-labeled G-box-containing probe; –, absence of the relevant probes or proteins; +, presence of the relevant probes or proteins.
